# Vimentin regulates differentiation switch via modulation of keratin 14 levels and their expression together correlates with poor prognosis in oral cancer patients

**DOI:** 10.1371/journal.pone.0172559

**Published:** 2017-02-22

**Authors:** Crismita Dmello, Sharada Sawant, Hunain Alam, Prakash Gangadaran, Saie Mogre, Richa Tiwari, Zinia D’Souza, Manish Narkar, Rahul Thorat, Komal Patil, Devendra Chaukar, Shubhada Kane, Milind Vaidya

**Affiliations:** 1 Cancer Research Institute (CRI), Advanced Centre for Treatment, Research and Education in Cancer (ACTREC), Tata Memorial Centre (TMC), Kharghar, Navi Mumbai, India; 2 Homi Bhabha National Institute, Training school complex, Anushakti Nagar, Mumbai, India; 3 Surgical Oncology, Head and Neck Unit, Tata Memorial Hospital (TMH), Parel, Mumbai, India; 4 Department of Pathology, Tata Memorial Hospital (TMH), Parel, Mumbai, India; Medizinische Fakultat der RWTH Aachen, GERMANY

## Abstract

Vimentin is an intermediate filament protein, predominantly expressed in cells of mesenchymal origin, although its aberrant expression is seen in many carcinomas during epithelial mesenchymal transition. In cancer, vimentin expression is associated with the transition from a more differentiated epithelial phenotype to a dedifferentiated state. In view of the perceived role of keratins (Ks) as regulators of differentiation in epithelia, it was important to understand whether vimentin modulates differentiation through the reprogramming of keratins, in transformed cells. To address this, vimentin was stably downregulated in oral cancer derived cells. Further, global keratin profiling was performed after high salt keratin extraction. K5/K14 pair was found to be significantly downregulated, both at protein and mRNA levels upon vimentin downregulation. The previous study from our laboratory has shown a role of the K5/K14 pair in proliferation and differentiation of squamous epithelial cells. Vimentin depleted cells showed an increase in the differentiation state, marked by an increase in the levels of differentiation specific markers K1, involucrin, filaggrin and loricrin while its proliferation status remained unchanged. Rescue experiments with the K5/K14 pair overexpressed in vimentin knockdown background resulted in decreased differentiation state. ΔNp63 emerged as one of the indirect targets of vimentin, through which it modulates the expression levels of K5/K14. Further, immunohistochemistry showed a significant correlation between high vimentin-K14 expression and recurrence/poor survival in oral cancer patients. Thus, in conclusion, vimentin regulates the differentiation switch via modulation of K5/K14 expression. Moreover, vimentin-K14 together may prove to be the novel markers for the prognostication of human oral cancer.

## Introduction

Vimentin is normally a mesenchymal-specific, type III intermediate filament (IF) protein. Traditionally, vimentin has been known to maintain cellular integrity and provide resistance to stress [1]. Studies on mouse embryos have shown that, during embryogenesis, synthesis of vimentin takes place for the first time exclusively in the primary mesenchymal cells at the primitive streak stage [2]. Moreover, vimentin is replaced partially or completely with their cell type specific IF protein in the terminally differentiating neuroepithelial cells [3]. Also, transgenic mice overexpressing the vimentin gene showed an inhibition in the normal differentiation of lens fibers [4]. Even in the liver development, expression of vimentin is associated with an undifferentiated status of liver cells [5]. Consistent with this, vimentin has been shown to impede osteoblast terminal differentiation by inhibiting the transactivation function of activating transcription factor 4 (ATF4) [6]. Furthermore, vimentin expression was found to correlate well with the poorly-differentiated and fibroblastic phenotype of breast cancer cell lines [7]. The above evidence suggests the ability of vimentin to retain dedifferentiation, most likely by restricting differentiation.

Vimentin expression has recently gained importance from the point of view of identifying the mesenchymal origin of a cell, as a prognostic marker to predict the biology of the tumor and to detect micro-metastasis [8]. Overexpression of vimentin is seen in various carcinomas like prostate, gastrointestinal, breast, lung and also melanomas [9]. Our laboratory along with others has shown a correlation of aberrant vimentin expression with lymph node metastasis, recurrence and also poor survival [10–12]. Vimentin is now being perceived not only as a canonical marker but also as a driver of epithelial–mesenchymal transition (EMT) [8]. During the process of EMT, the cell acquires a characteristic flattened morphology (mesenchymal-like) due to loss of cell-cell and cell-substrate contact. At the same time, there is a dramatic reprogramming of epithelia-specific keratins, to initiate the expression of mesenchyme-specific protein vimentin [13]. Such a change in keratin composition, associated with a reduction in the degree of differentiation, is also seen during an injury (inflammation/atrophy) wherein a cell starts expressing more than two keratins/IF proteins, e.g. K7, K17, K19, vimentin etc.[14]. Recent evidence of cytoskeletal remodeling was seen in the case of mouse embryonic fibroblasts, in the course of transition between progenitor and differentiated states [15]. Due to tissue and differentiation state-specific expression of keratins, many of them have found wide utility in clinics as diagnostic markers especially in tumor pathology. Expression patterns of K5, K6, K14, K17, etc. have been shown to positively correlate with the dedifferentiated and metastatic nature of squamous cell carcinomas [16]. Our group has shown aberrant expression of K8/K18 and its functional/molecular role in the progression of oral squamous cell carcinoma (OSCC) [17, 18].

Previous work from our laboratory has shown the contribution of K5/K14 pair in the maintenance of a proliferative and dedifferentiated state of the tongue squamous cell carcinoma (SCC) derived cell line AW13516 [19]. In the current study, we found a reduction in the expression of K5/K14 pair upon vimentin downregulation, perhaps mediated through ΔNp63, resulting in a more differentiated phenotype of the tumor cell. Thus, this study depicts the modulatory role of vimentin to fine tune the differentiation switch to favor tumor progression.

## Materials and methods

### Ethics statement

This study was approved by the “Human Ethics Committee”, Tata memorial centre, India (Reg Number: DCGI: ECR/149/Inst/MH/2013) and the written “informed consent” was obtained from all the patients before enrolling them in this study. All protocols for animal studies were reviewed and approved by the ‘‘Institutional Animal Ethics Committee (IAEC)” constituted under the guidelines of the ‘‘Committee for the Purpose of Control and Supervision of Experiments on Animals (CPCSEA)”, Government of India (Approval ID: 19/2014).

### Housing and monitoring of experimental animals

NOD-SCID mice used in the present study were procured from Charles River Laboratory UK Ltd. Mice were maintained under strict specific pathogen-free (SPF) conditions and all animals used for this study were healthy. Animals were housed in Individually Ventilated Caging system (M/S Citizen Industries, Ahmedabad, Gujrat, India) and provided with commercially available corn cob bedding material from ATNT Laboratories, Mumbai MS, India. Overall dimensions of the IVC cage are L 369 mm x W 156 mm x H 182 mm; cage floor area is 360 cm^2^. The animals were housed in a controlled environment under 23±2°C, 40–70% relative humidity and the dark-light cycle of 12 h each. The animals received sterile water *ad libitum* and autoclaved balanced diet prepared in-house from natural ingredients like wheat, roasted Bengal gram, casein, milk powder, ground nut oil, vitamins and mineral supplements which provide approximately 21% crude protein. As a quality control program, CD4, CD8 and CD19 status of NOD-SCID animals are checked, using flow cytometry. This is performed twice a year with randomly selected animals from the expansion colony. Periodically, the microbiological and clinico-pathological status of animals is also tested. Prior to the injection of tumor cells, no anesthetics or analgesics were given to the animals, in order to alleviate momentary pain. Also, the animals were not euthanized prior to the injection of tumor cells. During the injections, the mice were handled by a trained, certified animal technician and the cells were injected into the dorsal flank region by the in-house attending veterinarian (FELASA certified), ensuring minimum distress. After 42–52 days post injection, the animals were sacrificed so that the mean tumor diameter did not exceed 1.2 cm, as described in the AAALAC guidelines for animal welfare in cancer research [20]. Animal injury/illness or mortality was not seen during the course of this study. Every investigator follows the institute’s protocol (displayed on the institute’s intranet) to set humane endpoints prior to the sanction by the IAEC. For euthanization, the mice were kept in a chamber (which was vented out before the next euthanasia) for 2’-3’ and then CO_2_ was introduced from the cylinder supply valve into the chamber at an optimal flow rate of 20% of the chamber volume. After verifying the cessation of respiration and heart beats, cervical dislocation was performed by skilled personnel to confirm the death.

### Cell lines, antibodies and reagents

The establishment and characterization details of AW13516, AW8507 [21], DOK [22], HaCat [23] and A431 [24] are as described previously. Lists of cell lines, antibodies and reagents with their particulars are given in the S1–S3 Tables respectively.

### Plasmids and retroviral constructs

The details of the generation of vimentin knockdown and vector control clones are as described previously [25]. For vimentin overexpression, emerald GFP-vimentin retroviral construct (a kind gift from Professor Robert Goldman) was used. Emerald GFP-K14 construct was generated by amplifying K14 gene sequence from K14-pEGFP-N3 construct and subcloned into emerald GFP-pQCXIP vector using the single BamHI site. Firstly, emerald GFP-K14 was overexpressed in vimentin knockdown background shvim2 and the positive clones were sorted using FACSAria based on the GFP fluorescence. Next, the K14 positive clones were used to transduce K5 pLNCX2 retroviral construct (a kind gift from Professor Thomas Magin) and the positive clones were selected using G418 sulphate (400μg/ml). The empty vector backbone of both the constructs was used to generate the vector control clones. For the cloning of ΔNp63α, its cDNA was prepared from HaCat cell line and was cloned into the pLNCX2 vector containing N-terminal flag tag sequence using HindIII and SalI restriction enzyme sites. Flag-ΔNp63α containing retroviral construct was then transduced into vimentin knockdown clone shvim2 and positive clones were selected using G418 sulphate (400μg/ml). The empty flag pLNCX2 vector backbone served as a vector control.

### Quantitative Real-Time PCR (qRT-PCR) and Reverse Transcriptase PCR (RT-PCR)

RNA was isolated with the TRI reagent and cDNA was prepared using Revert Aid First Strand cDNA synthesis Kit according to the manufacturer’s protocol. qRT-PCR and RT-PCR were performed as described previously [26]. The list of qRT-PCR and RT-PCR primer sequences is given in the S4 Table. Primer sequences used for the detection of ΔNp63 isoforms were adapted from a report by Sniezek et al. [27].

### Western blotting, high salt keratin extraction, two-dimensional (2D) gel electrophoresis and mass spectroscopy

Western blotting was performed as described previously [17]. Briefly, whole-cell lysates were prepared in SDS lysis buffer (62.5 mM Tris (pH 6.8), 2% SDS, 0.1% BME (β -Mercaptoethanol) and 10% glycerol). A protease inhibitor cocktail was added to the lysis buffer. An equal amount of protein was loaded and resolved on SDS–PAGE gels followed by western blotting. Keratin enrichment was done using a high salt extraction protocol described previously [28]. This high salt keratin-rich fraction was subjected to 2D gel electrophoresis using IPG strips (pH 3–10). The gel was then stained with coomassie brilliant blue and differentially expressed spots were excised and processed for matrix-assisted laser desorption ionization (MALDI) analysis as previously described [18].

### Preparation of cytoplasmic and nuclear fractions

The cytoplasmic fraction was separated using 1X hypotonic cell lysis buffer (along with protease and phosphatase inhibitors). The hypotonic cell lysis buffer was prepared as per the composition given in the manual of CelLytic NuCLEAR Extraction Kit (Sigma, product code NXTRACT). The cytoplasmic fraction was separated and the nuclear pellet was resuspended in SDS lysis buffer, vortexed, boiled for 5’ and centrifuged at 13,000 rpm for 20’. The supernatant obtained was used as the nuclear fraction.

### Immunofluorescence

For immunofluorescence, cells were grown on coverslips for 48 h and treated with 0.03% Triton X-100 in chilled methanol for 90 seconds. Permeabilized cells were then fixed in chilled methanol for 10’ at -20°C. Further, the procedure followed is as described previously [17]. All confocal images were acquired using Zeiss LSM 780 microscope.

### Cell proliferation and clonogenic assays

Cell proliferation using MTT (3-(4,5-dimethylthiazol-2-yl)-2,5-diphenyltetrazolium bromide) assay was performed as per the previously described protocol [19]. Accordingly, 1000 Cells were seeded per well in triplicates and monitored over a period of 4 days. The absorbance of the well was considered as a measure of cell density to study the proliferation capacity of the clones. For the clonogenic assay, 200 cells of each clone were seeded in 60mm dishes and monitored for 10 days till visible colonies were formed. The colonies were then fixed in methanol for 5’ and stained with 0.5% crystal violet (prepared in 20% methanol) for 5’.

### Tumorigenicity assays

The tumorigenic potential of vimentin knockdown/vector control and K5/K14 overexpressing/vector control clones was determined by subcutaneous injection into NOD-SCID mice. Six mice were injected per clone (6 × 10^6^ cells each) and were observed for tumor formation over a period of approximately two months. Tumor dimensions were determined using a vernier caliper and its volume was calculated according to the modified ellipsoidal formula, [Tumor volume = 1/2(length × width^2^)] [29].

### Immunohistochemistry (IHC) and statistical analysis

IHC was performed on the OSCC tissue samples (48 SCCs of the tongue and 52 SCCs of the buccal mucosa) collected from the operation theatre of Tata Memorial Hospital, India. IHC analysis was also performed on mice tumor tissues that originated from vimentin knockdown-vector control and K5/K14 overexpressing-vector control groups. Statistical analysis was carried out as described previously [26]. A *p* value less than 0.05 was considered statistically significant. The clinico-pathological data and mice tumor volumes were analyzed using Statistical Package, SPSS 16.0 and SPSS 21.0 respectively.

## Results

### Downregulation of vimentin results in an alteration in the keratin profile of the OSCC derived cell line AW13516

**Fig 1:**
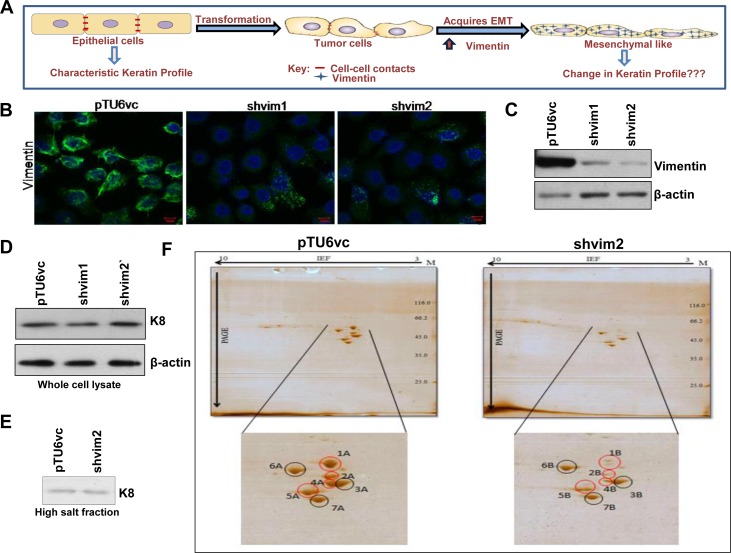
Downregulation of vimentin resulted in a change in the global keratin profile of the oral SCC derived cell line AW13516. (A) Schematic representation of the hypothesis. As a tumor cell acquires EMT (marked by upregulation of vimentin), it undergoes transition from a more epithelial-like to a more mesenchymal-like dedifferentiated state. To achieve this transition, there could be a vimentin mediated reprogramming of the keratins which distinguish these states. (B and C) Immunofluorescence (Bar: 10 μm) and western blot analysis of vimentin knockdown (shvim1 and shvim2) and its vector control clone (pTU6vc) using an antibody against vimentin. β-actin was used as the loading control in the western blotting experiment. (D) K8 levels of vimentin knockdown and its vector control clones were analyzed using western blotting. β-actin was used as a loading control. (E) The expression of K8 does not change upon vimentin downregulation. Thus, K8 was used as the loading control for high salt keratin enriched fraction. (F) Representative images of the 2D-gel, which show changes in keratin expression in the high salt keratin enriched fractions of vimentin knockdown and its vector control clones. The black circles indicate similarly expressed while the red circles indicate differentially expressed proteins. All the experiments were repeated independently in triplicates. For all the western blot experiments, the numbers below each blot represent the relative intensity of the bands determined using densitometry.

**Fig 2:**
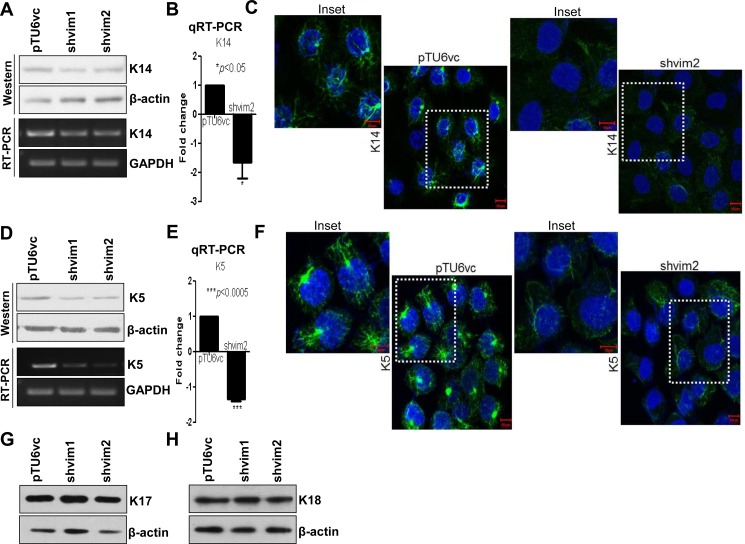
Vimentin knockdown cells show downregulation of both K5 and K14 at the mRNA as well as protein level. (A and B) Western blot, RT-PCR and qRT-PCR respectively of K14, in vimentin knockdown and vector control clones. (C) Immunofluorescence analysis (Bar: 10 μm) of the K14 levels in the indicated clones. Insets display magnification of the K14 filaments (in the adjacent figure of the one marked by the dotted white boxes). (D and E) Western blot, RT-PCR and qRT-PCR respectively of K5, in vimentin knockdown and vector control clones. (F) Immunofluorescence analysis (Bar: 10 μm) of the K5 levels in the indicated clones. Insets display magnification of the K5 filaments (in the adjacent figure of the one marked by the dotted white boxes). (G and H) Western blot analysis demonstrates unchanged levels of K17 and K18 in vimentin knockdown as compared to vector control clones. β-actin was used as the loading control in all the western blotting experiments. GAPDH was used as the loading control in RT-PCR. For qRT-PCR experiments; the relative expression of the target genes was normalized to GAPDH. The graphical data represents ± standard error mean (SEM) of three independent experiments.

Expression of vimentin is strongly associated with the characteristic phenotype of the cells undergoing EMT. Hence, we hypothesized that vimentin may not only be a marker but also a molecular regulator involved in reprogramming the expression of keratins to transit from a differentiated to a more dedifferentiated state (Fig 1A). In order to test this hypothesis, we used vimentin knockdown clones generated in the OSCC derived cell line AW13516 [25]. Downregulation of vimentin was confirmed by immunofluorescence and western blotting analysis (Fig 1B and 1C) and (S1 Fig). To identify differentially regulated keratins, high salt keratin extraction followed by 2D and MALDI analysis was performed using vimentin knockdown and vector control clones. K8 was used as the loading control for high salt extracted fractions since its levels did not alter upon vimentin downregulation (Fig 1D–1F) and (S1 Fig). The global keratin profile revealed the identity of differentially expressed proteins across the clones, one among which was found to be K14, along with the known differentially expressed protein, vimentin (Table 1). The appearance of actin (though it is easily soluble in mild buffers) in the high-salt extracted fraction could be attributed to its high abundance in the cell. Downregulation of K14 at the protein level was confirmed by western blotting and immunofluorescence analysis. Interestingly, transcript level analysis showed decreased expression of K14 at the mRNA level itself (Fig 2A–2C and S2 Fig). Furthermore, the binding partner of K14, which is K5, was also found to be downregulated both at the protein and mRNA level in vimentin knockdown clones (Fig 2D–2F and S2 Fig). This suggests that vimentin may modulate the expression of K5/K14 by unknown mechanism/s. However, since the downregulation of K5/K14 pair was at the transcript level itself, the possibility of altered solubility was excluded. The levels of K17 and K18 remained unchanged between the clones, as determined using western blot analysis, which validated the 2D gel observations (Fig 2G and 2H and S2 Fig). Hereafter, for all the experiments, vimentin knockdown clone shvim2 was used, which showed a higher degree of vimentin downregulation as compared to shvim1.

**Table 1:** List of proteins identified using MALDI analysis.

Spot	Protein	Mass	pI	Score	Matched Peptides	Total Peptides		Protein Sequence Coverage	Expression status of proteins in vimentin knockdown as compared to vector control clone
1A	VIME_HUMAN (Vimentin, OS = Homo sapiens)	53676	4.9	74	8	39		23.6%	Downregulated
2A	Keratin 14	51872	5.09	52	10	50		23%	Downregulated
3B	K1C17_HUMAN (Keratin Type 1, Cytoskeletal 17, OS = Homo sapiens)	48361	4.8	156	15	26		50.9%	Unchanged
4A	Unidentified								Downregulated
5A	K1C18_HUMAN (Keratin, Type I Cytoskeletal 18 OS = Homo sapiens)	48029	5.2	76	17	60		51.4%	Unchanged
6A	K2C8_HUMAN (Keratin Type 2, Cytoskeletal 8, OS = Homo sapiens)	53671	Predicted as K8 but with low score						Unchanged
7A	ACTB_HUMAN (Actin, Cytoplasmic 1 and 2, OS = Homo sapiens)	42052	5.2	75	7	57	38.9%		Unchanged

MALDI analysis: The resulting data of the keratin spots was analyzed using Flex analysis 3.0 (BruckerDaltonik, Germany) software. The peak list was searched against SwissProt database using MASCOT search engine. The last column shows the expression status of proteins in vimentin knockdown as compared to its vector control clone after validating the spots identified by MALDI, with western blotting.

### Vimentin modulates the differentiation status and tumorigenic potential of epithelial cells

**Fig 3:**
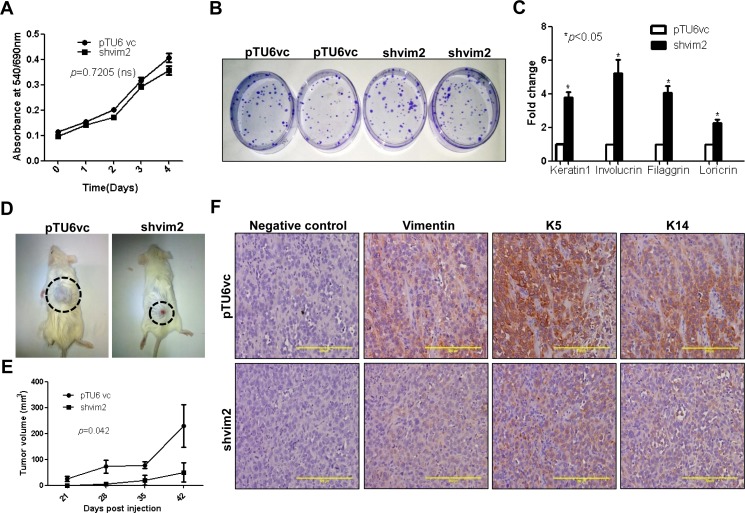
Phenotypic alterations associated with K5/K14 downregulation as a consequence of vimentin depletion. (A) Proliferation curves of vimentin knockdown and its vector control clones over a period of 4 days, using MTT assay. (B) Representative image of the clonogenic assay shows colonies formed by vimentin knockdown and its vector control clones. (C) qRT-PCR analysis of differentiation specific markers K1, involucrin, filaggrin and loricrin respectively. The relative expression of the target gene was normalized to GAPDH. For (A and C), the graphical data represents ± SEM of three independent experiments. (D) Representative images of tumorigenicity assays using NOD-SCID mice (6 animals each) injected with either vimentin knockdown or vector control clones. The tumors are indicated by dotted circles. (E) The tumor measurements were recorded up to 42 days, after which the animals were sacrificed and the tumor tissue was isolated for IHC staining. The graph shows the tumor volume plotted against time for both the clones. (F) Representative images (Bar: 200μm) of IHC staining for expression of vimentin, K5 and K14 respectively in mice tumor tissues. The negative control images represent tissue sections incubated with serum from non-immunized mice in place of primary antibodies.

The K5/K14 expression is a typical feature of progenitor basal stem cells of stratified epithelial origin. Also, a previous report from our laboratory has shown direct evidence wherein the K5/K14 pair is able to regulate cell proliferation, differentiation and neoplastic progression in the same system AW13516 [19]. Surprisingly, MTT assay did not show any obvious difference in the proliferation potential of vimentin knockdown as compared to its vector control cells (Fig 3A). In addition, we performed a clonogenic assay on vimentin knockdown and vector control clones. Again, no significant difference was seen in the number and size of colonies between the clones which correlated with their similar proliferation rates (Fig 3B). Further staining with cell proliferation markers, proliferating cell nuclear antigen (PCNA) and Ki67 confirmed our results (S3A and S3B Fig). However, during epithelial stratification, as a basal cell gets committed to differentiation, it loses the expression of K5/K14 pair and starts expressing involucrin, filaggrin, loricrin and differentiation specific keratins in the upper layers [30]. Vimentin downregulation led to an increased expression of K1, involucrin, filaggrin and loricrin respectively (Fig 3C) and involucrin protein levels (S4 Fig) as determined using qRT-PCR and western blotting. However, transcript levels of multipotent stem cell marker Oct-4 (S5 Fig) decreased significantly. Further, to assess the tumorigenic role of vimentin, vimentin knockdown and vector control cells were injected subcutaneously into NOD-SCID mice (Fig 3D). A significant reduction was observed in the tumor volume in vimentin knockdown as compared its vector control group (Fig 3E). Furthermore, IHC analysis confirmed the decrease in the expression of K5/K14 (Fig 3F) and increase in the expression of involucrin (S6 Fig) in tumors formed in mice from vimentin knockdown cells. Moreover, expression of PCNA did not show any difference among the two groups (S6 Fig). Collectively, these results suggest that vimentin is involved in maintaining a more dedifferentiated state of the cancer cell, perhaps through modulation of K5/K14 expression.

### Vimentin positively regulates the expression of K5/K14 pair across different stratified epithelial-derived cell lines

**Fig 4:**
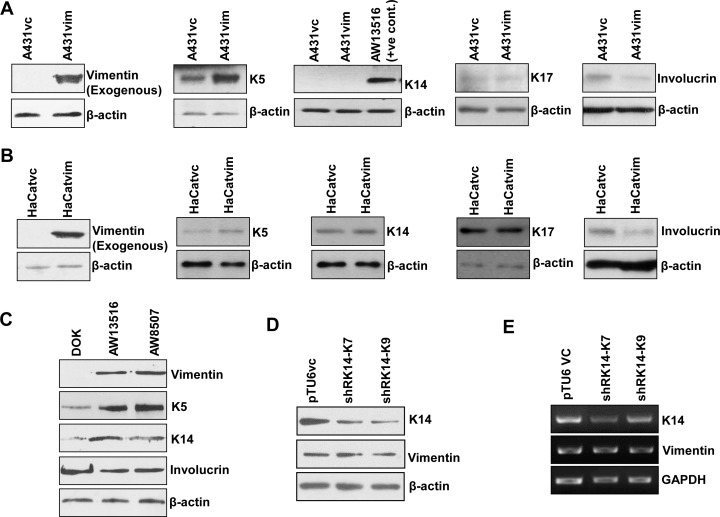
Vimentin mediated positive regulation of K5/K14 levels is not a cell line specific phenomenon. (A and B) Western blot analysis shows protein level of vimentin, K5, K14, K17 and involucrin in vimentin overexpressing clones of A431vim and HaCatvim as compared to its respective vector control clones A431vc and HaCatvc. Since A431 does not express K14, whole cell lysate from AW13516 was used as a positive control. (C) Whole cell lysates from DOK, AW13516 and AW8507 cells were probed with antibodies against vimentin, K5, K14 and involucrin respectively using western blotting. (D and E) Western blot and RT-PCR analysis of K14 and vimentin in K14 knockdown (shRK14-K7 and shRK14-K9) and its vector control clones (pTU6-AW1). GAPDH was used as the loading control in RT-PCR experiment. β-actin was used as the loading control in western blotting experiments. Western blotting experiments were done thrice with three independent sets of samples.

To verify whether the regulation of K5/K14 expression by vimentin is not a cell line specific phenomenon or an off-target effect of the shRNA associated vimentin downregulation, it was overexpressed in vimentin lacking, A431 and HaCaT cell lines. Vimentin upregulation in A431 cells led to a concomitant increase in the levels of K5, but a marked decrease was seen in the levels of differentiation specific protein, involucrin [31]. However, since A431 cells do not express K14, we checked for the levels of K17, considering the fact that K17 is known to pair with K5 in the absence of K14 [32, 33]. We found no change in the levels of K17 upon vimentin overexpression, which further confirmed that the regulation exerted by vimentin on the expression of K5/K14 is highly specific (Fig 4A and S7A Fig). Forced expression of vimentin in HaCaT also showed a marked increase in the expression of K5 while K14 expression was unaffected. The differentiation specific marker involucrin showed a significant decrease in its levels, in response to an increase in the expression of vimentin (Fig 4B and S7B Fig). Next, we assessed the status of K5/K14 and involucrin with respect to vimentin in some tongue derived cell lines. High vimentin expressing AW13516 and AW8507 cells (both of which are SCC derived cell lines) displayed elevated protein levels of K5/K14 and decreased levels of involucrin as compared to DOK cells (dysplastic lesion derived cell line), which does not express vimentin (Fig 4C and S7C Fig). Additionally, to determine whether K14 also has a similar regulatory effect on vimentin, we assessed the levels of vimentin in the same system AW13516, with the K14 knockdown background. Both the protein and mRNA levels of vimentin remained unchanged (Fig 4D and 4E and S7D Fig), suggesting that vimentin may be upstream in pathway/s that regulates the expression of K14.

### Vimentin knockdown phenotype was rescued upon re-expression of K5/K14 pair

**Fig 5:**
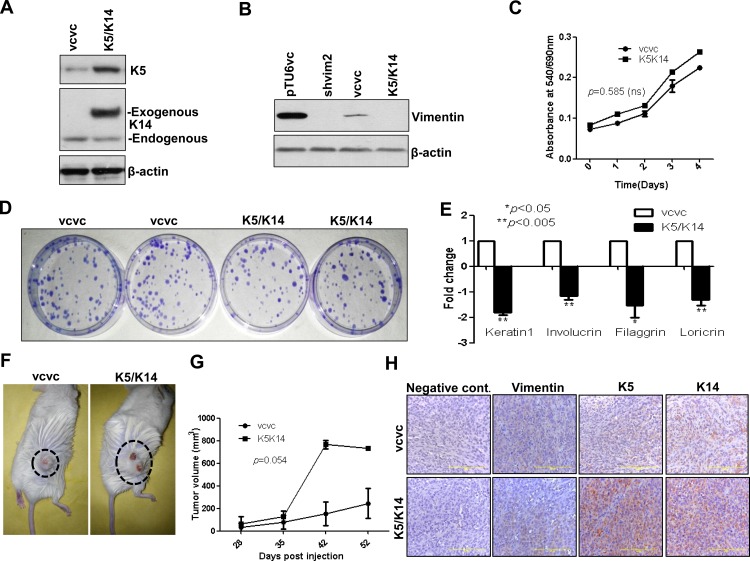
Vimentin knockdown phenotype was rescued upon re-expression of K5/K14 together, in vimentin knockdown background. (A and B) Western blot analysis shows overexpression of emerald GFP-K14 and K5 in K5/K14 overexpressing clone (“K5/K14”-shvim2 transduced with emerald GFP-K14-pQCXIP and K5-pLNCX2 overexpressing vectors) as compared to its vector control clone (“vcvc”-shvim2 transduced with emerald GFP-pQCXIP and pLNCX2 empty vectors). Protein levels of vimentin were tested in vimentin knockdown-vector control and K5/K14 overexpressing-vector control groups to confirm the maintenance of vimentin knockdown background in the second group, using western blotting. (C) Proliferation curves of K5/K14 overexpressing and its vector control clones over the period of 4 days, using MTT assay. (D) Representative image of clonogenic assay shows colonies formed by K5/K14 overexpressing and its vector control clones. (E) qRT-PCR analysis of differentiation specific markers K1, involucrin, filaggrin and loricrin respectively. The relative expression of the target gene was normalized to GAPDH. (F) Representative images of tumorigenicity assays using NOD-SCID mice (6 animals each) injected with either K5/K14 overexpressing or its vector control clones. The tumors are indicated by dotted circles. (G) The tumor measurements were recorded up to 52 days, after which the animals were sacrificed and the tumor tissue was isolated for IHC staining. The graph shows tumor volume plotted against time for both the clones. (H) Representative images (Bar: 200 μm) of IHC staining for expression of vimentin, K5 and K14 respectively in mice tumor tissues. The negative control images represent tissue sections incubated with serum from non-immunized mice in place of primary antibodies.

Next, we re-expressed the K5/K14 pair in vimentin knockdown background because of two reasons: 1. to verify if the changes associated with vimentin knockdown are specifically due to K5/K14 downregulation and 2. To exclude the possibility of involvement of other altered molecule/s. Western blot (Fig 5A and 5B, S8A–S8C Fig) and immunofluorescence analysis (S9A and S9B Fig) confirmed the overexpression of K5K14 pair in the shvim2 clone (shvim2-vimentin knockdown clone was used, since it-showed a higher degree of vimentin downregulation as compared to shvim1 clone). As expected, proliferation and clonogenic potential remained unchanged upon K5/K14 re-expression, which corroborated with that of the vimentin knockdown phenotype (Fig 5C and 5D). K5/K14 re-expressing clone (“K5/K14”-shvim2 transduced with emerald GFP-K14-pQCXIP and K5-pLNCX2 overexpressing vectors) showed a decrease in the expression of differentiation specific markers K1, involucrin, filaggrin and loricrin (Fig 5E) and in the involucrin protein levels (S10 Fig) as determined using qRT-PCR and western blotting respectively. However, stemness marker Oct-4 was upregulated (S11 Fig) in K5/K14 re-expressing clone as compared to its vector control (“vcvc”-shvim2 transduced with emerald GFP-pQCXIP and pLNCX2 empty vectors). Interestingly, no significant change was observed in the levels of involucrin upon either K5 (S12 Fig) or K14 (S13 Fig) overexpression alone, indicating that perhaps both are required for conferring a more de-differentiated phenotype to the tumor cell. Tumorigenic potential of K5/K14 re-expressing clones was also significantly higher as reflected by the increased subcutaneous tumor growth in NOD-SCID mice (Fig 5F–5H). Further, tumors formed in mice from K5/K14 re-expressing clones showed a decrease in the expression of involucrin while no change was observed in the expression of PCNA (S14 Fig). This rescue experiment suggests that vimentin mediates regulation of the differentiation switch via the reprogramming of basal cell specific K5/K14 expression.

### ΔNp63 could be a possible target of vimentin, to bring about the modulation of K5/14 expression

**Fig 6:**
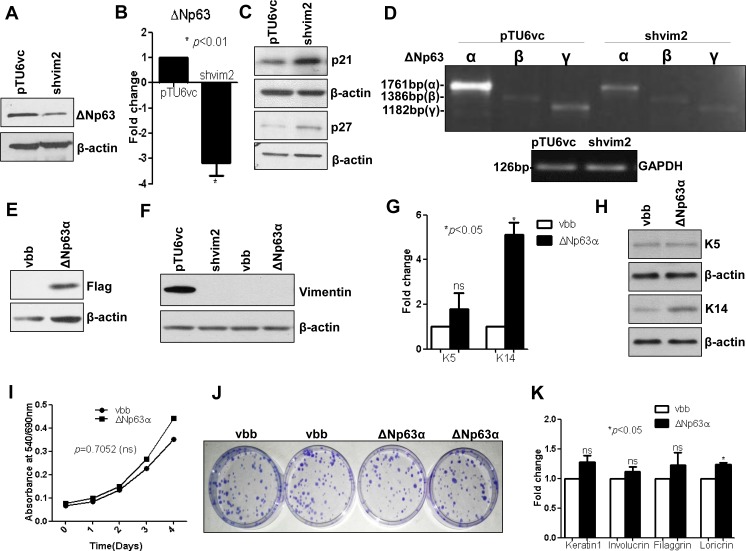
Vimentin knockdown phenotype was partially rescued upon re-expression of ΔNp63α in vimentin knockdown background. (A) Western blot analysis shows protein levels of ΔNp63 from whole cell lysates of vimentin knockdown and its vector control clones. (B) Fold change in mRNA expression level of ΔNp63 in vimentin knockdown as compared to vector control clones, using qRT-PCR analysis. (C) Western blot analysis shows protein levels of p21 and p27 from the whole cell lysates of vimentin knockdown and its vector control clones. (D) RT-PCR analysis shows expression of ΔNp63α, β and γ isoforms, between vimentin knockdown and vector control clones. GAPDH was used as a loading control. (E) Western blot analysis using anti-flag antibody confirmed the overexpression of flag-tagged ΔNp63α. (F) The protein level of vimentin was tested by western blotting in vimentin knockdown-vector control set and flag-ΔNp63α-vector control set, to confirm the maintenance of vimentin knockdown background in the second group. (G and H) qRT-PCR and western blot analysis of K5 and K14 in flag-ΔNp63α overexpressing and its vector control group. (I) Proliferation curves of flag-ΔNp63α overexpressing and its vector control clones over the period of 4 days, using MTT assay. (J) Representative image of clonogenic assay shows colonies formed by flag-ΔNp63α overexpressing and its vector control clones. (K) QRT-PCR analysis of differentiation specific markers K1, involucrin, filaggrin and loricrin. For all the qRT-PCR experiments, the relative expression of the target gene was normalized to the GAPDH. For all western blotting experiments, β-actin was used as a loading control. The graphical data represents ± SEM of three independent experiments.

Next, we wanted to investigate the molecular regulator, through which vimentin modulates the expression of K5/K14. ΔNp63 is known to directly regulate the expression of both K5 and K14, during the program of keratinocyte stratification [34, 35]. Hence, as a first step, we checked its levels in the vimentin knockdown background. Western blot and qRT-PCR analysis showed decreased levels of ΔNp63, both at protein and mRNA levels respectively (Fig 6A and 6B and S15A Fig). The antibody used for detection of ΔNp63 is a pan-p63 antibody (SC-8343), which recognizes α, β and γ isoforms of p63. Since the expression of ΔNp63α isoform is very abundant, it is very likely that the single band seen in western blot is of ΔNp63α [36]. To functionally characterize depletion of ΔNp63, we checked for other known molecular alterations associated with ΔNp63 loss. Vimentin knockdown clones showed increased levels of p21 and p27 (Fig 6C and S15A Fig), which are typically associated with ΔNp63α downregulation [37, 38]. Further, RT-PCR analysis showed downregulation of both ΔNp63α and ΔNp63γ isoforms in vimentin knockdown as compared to its vector control clones (Fig 6D). To verify whether the K5/K14 downregulation seen upon vimentin knockdown is due to reduced ΔNp63 levels, flag- tagged ΔNp63α (since ΔNp63α is a major isoform expressed in keratinocytes [37]) was stably re-expressed in vimentin knockdown clone shvim2 (Fig 6E and 6F and S15B and S15C Fig). ΔNp63α overexpression led to a significant increase in the levels of K14 while K5 expression was only marginally rescued, indicative of the contribution of more than one molecule in the regulation of K5/K14 expression (Fig 6G and 6H and S15D Fig). Nevertheless, K14 formed filaments despite the lesser levels of its partner K5 (S16A and S16B Fig), most likely due to the presence of K8 in AW13516 cells.[39]. The proliferation and clonogenic potential remained unaffected upon ΔNp63α overexpression (Fig 6I and 6J). The differentiation status (defined by the expression of differentiation specific markers) remained unchanged (Fig 6K) while the expression of Oct-4 showed only a marginal increase (not significant) (S17 Fig) upon ΔNp63α upregulation in the vimentin knockdown background.

### Notch1-ΔNp63 crosstalk may be involved in the vimentin mediated modulation of the differentiation switch

ΔNp63 and notch regulate each other by a negative feedback loop [37]. In order to understand the cause of ΔNp63 downregulation, we checked for the levels of activated Notch1 upon vimentin knockdown. Vimentin knockdown clone showed increase in activated Notch1 (notch intracellular domain (NICD)) levels as compared to the vector control clone (S18A Fig and S19A Fig). The other candidate molecule which may modulate the levels of ΔNp63 is nuclear factor-kappaB (NF-κB), since it negatively regulates ΔNp63 either through the notch or independently as a part of the differentiation program [40]. Increased nuclear localization of NF-κB (p65) was observed in vimentin knockdown cells (S18B and S18C Fig and S19B Fig). On the other hand, these molecular changes showed reversal (decrease in activated Notch1 levels and in nuclear localization of NF-κB) upon ΔNp63α overexpression in vimentin knockdown background (S18D–S18F Fig and S19A and S19B Fig). Further, qRT-PCR analysis for Hes1 (which is a known Notch dependent target gene [41, 42]) suggested increase in notch activity upon vimentin depletion while the reverse was seen upon ΔNp63α overexpression in vimentin knockdown background (S19C Fig). Also, qRT-PCR analysis of IκBα (which is a known NF-κB dependent target gene [43, 44]) suggested increase in p65 activity upon vimentin depletion while the reverse was seen upon ΔNp63α overexpression in vimentin knockdown background (S19D Fig). Thus, our preliminary observations speculate the possibility of the crosstalk between notch (perhaps in an NF-κB dependent manner) and ΔNp63, to regulate differentiation state in vimentin knockdown cells.

### High expression of vimentin-K14 together correlates with recurrence and poor survival of oral cancer patients

**Fig 7:**
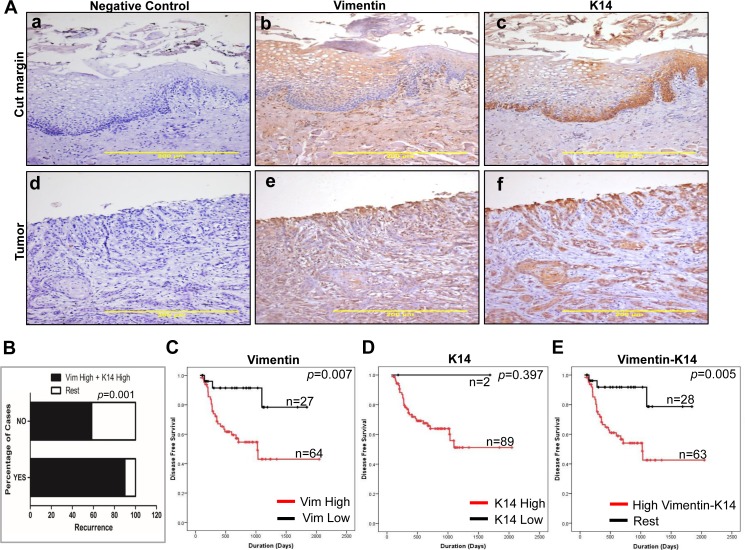
High vimentin-K14 expression correlates with poor survival in oral cancer patients. (A) (a-c) The upper panel show images (Bar: 200μm) of IHC staining for vimentin and K14 expression in cut margin tissues while lower panel (d-f) show images (Bar: 200μm) of IHC staining for vimentin and K14 expression in tumor tissues. The negative control images represent tissue sections incubated with serum from non-immunized mice in place of primary antibodies. (B) The graphical representation shows recurrence with respect to high vimentin-K14 expression and recurrence with respect to the other combinations of vimentin-K14 expression. Kaplan-Meier survival analysis (n = 91) of (C) High vs. low vimentin expression (D) High vs. low K14 expression and (E) High vimentin-K14 vs. the other combinations of vimentin-K14 expression.

Statistical correlation between clinico-pathological parameters and vimentin-K14 expression is listed in S5 Table. IHC analysis of OSCC tissues showed a positive correlation between high vimentin-K14 staining intensity (Fig 7A) and recurrence (*p* = 0.001) (Fig 7B). In order to determine if the vimentin-K14 status of the tumor has any association with the survival of the oral cancer patients, Kaplan-Meier survival analysis based on IHC data was performed on 91 oral tumor samples. The analysis showed a significant correlation between high vimentin (*p* = 0.007) and poor disease-free survival (Fig 7C). High K14 expression (*p* = 0.397) did not show a significant correlation with the disease-free survival (Fig 7D). This could be because of only 2 cases showing low expression of K14. On the other hand, a significant correlation was seen between high vimentin-K14 expression (*p* = 0.005) and poor disease free survival (Fig 7E). Collectively, this suggests that the expression of vimentin-K14 together may prove useful for the prognostication of human oral cancer. Additionally, we performed IHC for the expression of K5 and differentiation specific marker K1, on the same tumor samples whose vimentin and K14 expression was determined previously. Immunohistochemistry analysis (S20A Fig) showed positive correlation between K5/K14 and vimentin expression (Spearman’s non-parametric correlation = 0.500, *p* = 0.001, n = 100) in oral tumor tissues while no significant correlation was seen between the expression of K1 and vimentin (Spearman’s non-parametric correlation = -0.044, *p* = 0.663, n = 100) in oral tumor tissues. Further, Kaplan-Meier survival analysis showed a trend (albeit not significant) between high vimentin-K5-K14-low K1 expression and poor disease free survival (p = 0.966) (S20B Fig). Also, significant correlation was found between high vimentin-K5-K14-low K1 expression and thickness (p = 0.019) of the tumor mass (S6 Table).

## Discussion

Expression of EMT marker vimentin is usually associated with a more mesenchymal-like and dedifferentiated state of the cancer cell [8]. On the other hand, in the case of stratified epithelia, expression of one pair of keratins is specific to one particular differentiation compartment and as the cell moves from one layer to another, the expression pattern of keratins also changes accordingly [45]. This suggests that keratins may have a regulatory role in epithelial differentiation as well [46]. Hence, we wanted to understand whether vimentin modulates a differentiation switch of a transformed cell by modulating the expression of keratins. Our study found the K5/K14 pair to be a novel indirect target of vimentin, through which it is able to confer a dedifferentiated phenotype to the cancer cell.

To understand the overall change in the keratin profile caused due to downregulation of vimentin, we performed a global keratin profile analysis using high salt enriched keratin fraction from vimentin knockdown and vector control cells. Our 2D followed by MALDI analysis identified K14 as one of the differentially expressed proteins upon downregulation of vimentin. Interestingly, its binding partner K5 was also found to be downregulated at the protein level. Further, transcript level analysis showed that mRNA levels of both K5 and K14 are downregulated in vimentin knockdown clones.

K5/K14 pair is typically expressed by the basal stem cell layer of the stratified epithelium. As the cell from this layer is committed to differentiation, it replaces the expression of K5/K14 with the expression of one or more differentiation specific keratin pairs e.g., K1 and K10 [45]. A previous study from our laboratory has demonstrated the role of K5/K14 in modulating phosphatidylinositol 3-kinase/Akt–mediated cell proliferation and/or Notch1-dependent cell differentiation, in stratified epithelia derived cells [19]. Our current study showed an increase in the differentiation status of the vimentin knockdown cells, while its proliferation status remained unaffected. This change in differentiation could be attributed to the decreased levels of K5/K14 since its reversal was seen upon re-expression of K5/K14 in vimentin knockdown background. Proliferation potential of the cells remained unchanged upon vimentin knockdown, perhaps due to remnant levels of K5/K14 or because of an unknown compensatory mechanism operating in this situation.

Progenitor/stem-like and differentiated state are at extreme ends of the differentiation spectrum. Ding et al. have reported the loss of Oct-4 expression during the differentiation of mouse embryonic stem cells [47]. Hence, we were curious to understand if our vimentin knockdown cells, which were more differentiated showed any indications of somatic cell reprogramming. To verify this, we checked the expression of Oct-4, which is the master regulator of stemness [48]. Our results demonstrated a decrease in the expression of Oct-4 with the decrease in the expression of K5/K14, while re-expression of K5/K14 rescued the decreased levels of Oct-4. Nevertheless, additional experiments involving study of Oct-4 dependent target genes will help to elucidate if decrease in Oct-4 transcript levels has any functional impact in vimentin knockdown background. Transcription factors like AP1 and NF-κB are implicated in the regulation of basal expression of K5/K14 pair. The activation of these transcription factors is also dependent on extracellular signaling molecules like hormones, vitamins and growth factors [49]. Nevertheless, the role of the epithelial-specific master regulator p63 is well known for its precise control over epithelial cell differentiation [50]. One of the ways in which p63 regulates the differentiation program is through the maintenance of basal specific and stage specific expression of K5/K14 pair [51]. p63 is expressed as the isoform with a transactivation domain called as TA isoform and as a isoform lacking this domain, which is known as the ΔN isoform [52]. ΔNp63 isoform has been shown to act as a dominant negative regulator of TAp63 and p53 [53], which also suggest its oncogenic potential. ΔNp63 (which is the highly expressed isoform of p63 in the basal layer of epithelial tissues) is known to transcriptionally activate genes such as suppressor of fused homologue (SUFU), homeobox C4 (HOXC4) and myelin protein zerolike 2 (MPZL2; also known as EVA1) along with K5/K14 genes [52]. In our study, vimentin knockdown resulted in downregulation of ΔNp63, while p21 and p27 levels were found to be upregulated. However, despite the upregulation of cyclin-dependent kinase (Cdk) inhibitors, p21 and p27, the proliferation status of vimentin knockdown clone remained unaltered. Similar observations were also reported by Zheng et al., wherein neither p21 nor p27 knockout mice showed any alterations in the proliferation of mouse gastrointestinal tract cells [54]. Similarly, some cancer cells are known to proliferate despite CDK2 inhibition [55], suggesting that perhaps correlation between p21 and p27 with the cell proliferation is context dependent. Further, analysis of transcript level expression of all the three ΔNp63 isoforms revealed downregulation of both ΔNp63α and ΔNp63γ and no change in the levels of ΔNp63β. Report by Romano et al. suggested a role of ΔNp63α and ΔNp63β isoforms in inducing basal markers and stratification [35]. Therefore, to understand if vimentin modulates expression of K5/K14 pair through ΔNp63α isoform, we overexpressed ΔNp63α isoform (since levels of ΔNp63β remained unaltered upon vimentin downregulation) in vimentin knockdown background. Overexpression of ΔNp63α resulted in an increase in the expression of K14, while the expression of K5 remained unaltered. As a result of this, the differentiated state of the cell (marked by the expression of differentiation specific proteins) remained unchanged. This indicated that ΔNp63γ, may as well, have a role in the regulation of the expression of K5, which was not compensated by the overexpression of ΔNp63α isoform alone. The possibility of the involvement of some other K5 specific transcription factor/s (enhancer or repressor), downstream of vimentin, cannot be ruled out.

Levels of ΔNp63α and notch play a decisive role to either maintain stemness or to proceed towards differentiation [37]. During the stratification process of epidermal tissue, notch suppresses p63 to favor differentiation, as evident by their opposing levels in the basal compartment [40]. Our study showed an increase in the levels of notch1 with decreased ΔNp63 expression in the vimentin knockdown clone. Correspondingly, a decrease in the level of notch1 was seen upon overexpression of ΔNp63α. This suggests that there may be a reciprocal negative regulatory mechanism between notch and ΔNp63 in order to govern differentiation in the presence and absence of vimentin. There may be two possibilities in this situation: first being either the deficiency of vimentin relieves the inhibition on notch1 which in turn suppresses the expression of ΔNp63 or the second possibility is that downregulation of vimentin decreases the expression of ΔNp63 relieving the negative regulatory effect on notch1 and leading to its activation. Interestingly, while the downregulation of ΔNp63 has been shown to result in a decrease in the expression of vimentin in esophageal squamous carcinoma [56], we report the reverse here i.e., downregulation of vimentin leads to decreased expression of ΔNp63. There are reports of vimentin regulating transcript levels of certain genes in the literature. For instance, Vuoriluoto et al. have shown the role of vimentin in regulating the expression of several genes associated with EMT and the basal-like phenotype, one of which is Axl (a receptor tyrosine kinase) in breast cancer-derived cell lines [57]. Nevertheless, both the possibilities need to be tested experimentally to ascertain the directionality of the crosstalk between ΔNp63 and notch1 in vimentin knockdown background.

Factors other than activated notch may also have a significant role to play in the regulation of ΔNp63. Along these lines, Flores et al., have shown inhibition of wild-type p53 by ΔNp63 [58]. Moreover, the parental cell line under study, AW13516 expresses mutant p53 (R273H) [59]. Hence, work in this direction will be required to ascertain if mutant p53 is the cause or consequence of an alteration in ΔNp63α expression. Furthermore, specific roles of microRNAs (miRs) [60, 61], Wnt, Hedgehog and EGFR [50] pathways if any, in the regulation of ΔNp63 expression in vimentin depleted condition, needs to be investigated.

We found a significant correlation between high vimentin-K14 expression and recurrence as well as the poor survival of oral cancer patients. This finding can be explained by our *in vitro* data using vimentin knockdown system. This showed that vimentin expression promotes events leading to increased dedifferentiation and tumorigenicity, wherein K5/K14 upregulation seems to be an intermediate event. Similar observations were made by Thomas et al., who have reported the association of vimentin-keratin co-expression with poor prognosis and tumor phenotype [62]. Together, this suggests that vimentin aids the aggressiveness of the tumor by contributing to the maintenance of a dedifferentiated state of the tumor cell.

**Fig 8:**
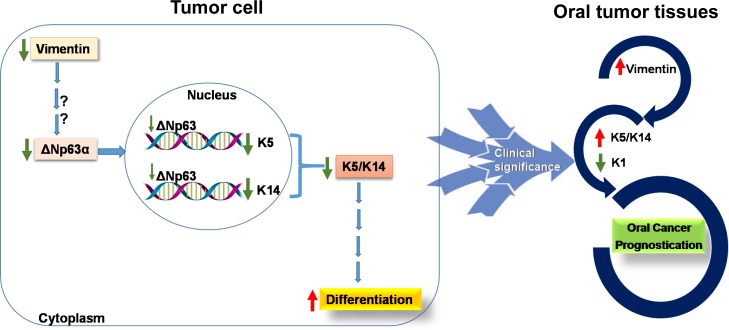
Schematic representation depicting the role of vimentin in modulation of K5/K14 expression, to regulate differentiation in carcinoma-derived cells. The model shows the regulation of K5/14 expression by vimentin, perhaps through ΔNp63. Determination of vimentin-K14 status in oral tumor tissues may have clinical implications.

In conclusion, our data sheds light on the modulatory role of vimentin in the expression of K5/K14 pair, to fine tune the differentiation switch in favor of tumor progression. Further, a large-scale study on human oral tumors is required to prove the potential of vimentin-K14 as prognostic markers for human oral cancer (Fig 8).

## Supporting information

S1 Fig Graph showing fold change in the protein level of vimentin and K8 upon vimentin downregulation. Graph shows quantitation of western blots using densitometry. Fold-change in vimentin and K8 protein level in vimentin knockdown clones is shown relative to that of its vector control clone. Error bars denote ± SEM. from three independent experiments.
(TIF)

S2 Fig Graph showing fold change in the protein level of K14, K5, K17 and K18 upon vimentin downregulation. Graph shows quantitation of western blots using densitometry. Fold-change in K14, K5, K17 and K18 protein level in vimentin knockdown clones is shown relative to that of its vector control clone. Error bars denote ±S.E.M. from three independent experiments.
(TIF)

S3 Fig Proliferation-related markers like PCNA and Ki67 remained unchanged upon vimentin downregulation. (A) Representative immunofluorescence images (Bar: 10μm) of PCNA (green) staining in vimentin knockdown and vector control clones. (B) Representative immunofluorescence images (Bar: 10μm) of Ki67 (red) staining in vimentin knockdown and vector control clones.
(TIF)

S4 Fig Vimentin downregulation led to a significant increase in the protein level of differentiation specific marker involucrin. (A) Western blot analysis shows protein level of involucrin from whole cell lysates of vimentin knockdown and its vector control clones. (B) Graph shows quantitation of western blot using densitometry. Fold-change in involucrin protein level in vimentin knockdown clone is shown relative to that of its vector control clone. Error bars denote ± SEM. from three independent experiments.
(TIF)

S5 Fig Oct-4 mRNA levels decreased significantly upon vimentin downregulation. QRT-PCR analysis of Oct-4 in vimentin knockdown and its vector control clones.
(TIF)

S6 Fig Tumors formed in mice from vimentin knockdown cells showed an increase in the expression of involucrin. Representative images (Bar: 200μm) of IHC staining for expression of involucrin and PCNA in tumor tissues of mice, injected with either vimentin knockdown or vector control clones. The negative control images represent tissue sections incubated with serum from non-immunized mice in place of primary antibodies.
(TIF)

S7 Fig Graphs showing fold change in the protein level of vimentin, K5, K14 and involucrin across different cell lines. Graphs show quantitation of western blots using densitometry. (A and B) Fold-change in vimentin, K5, K14, K17 and involucrin protein level in vimentin overexpressing clones of A431vim and HaCatvim is shown relative to its respective vector control clones A431vc and HaCatvc. (C) Fold-change in vimentin, K5, K14, K17 and involucrin protein level in AW13516 and AW8507 cells is shown relative to that of DOK cells. (D) Fold-change in K14 and vimentin protein level in K14 knockdown clones is shown relative to its respective vector control clone. Error bars denote ± SEM from three independent experiments.
(TIF)

S8 Fig Graphs showing fold change in the protein level of K5, K14 and vimentin upon K5/K14 re-expression in vimentin knockdown background. Graphs show quantitation of western blots using densitometry. (A and B) Fold-change in K5 and K14 protein level in K5/K14 overexpressing clone is shown relative to that of its vector control clone. (C) Fold-change in vimentin protein level in vimentin knockdown (shvim2), K5/K14 overexpressing clone (K5/K14) and its vector control clone (vcvc) is shown relative to that of vector control clone (pTU6vc). Error bars denote ± SEM from three independent experiments.
(TIF)

S9 Fig Confocal images showing filament organization of K5 and K14, after their re-expression in vimentin knockdown background. (A and B) Confocal microscopy analysis (Bar: 10μm) shows levels and filament networks of K5 and K14 respectively in K5/K14 (K5 and K14 overexpressing) as compared to its vector control vcvc clones (empty vectors of K5 and K14 together).
(TIF)

S10 Fig K5/K14 re-expression in vimentin knockdown background led to a significant decrease in the protein level of differentiation specific marker involucrin. (A) Western blot analysis shows protein level of involucrin from whole cell lysates of K5/K14 overexpressing as compared to its vector control clones. (B) Graph shows quantitation of western blot using densitometry. Fold-change in involucrin protein level in K5/K14 overexpressing clone is shown relative to that of its vector control clone. Error bars denote ± SEM from three independent experiments.
(TIF)

S11 Fig Oct-4 mRNA levels increased significantly upon K5/K14 re-expression in vimentin knockdown background. qRT-PCR analysis of Oct-4 in K5/K14 overexpressing and its vector control clones.
(TIF)

S12 Fig K5 re-expression alone in vimentin knockdown background failed to rescue the protein level of differentiation specific marker involucrin. (A) Immunofluorescence (Bar: 10 μm) images of K5 overexpressing (K5) and its vector control clone (K5vc) using antibodies against K5 and K14. (B) Western blot analysis shows protein level of K5, K14 and involucrin in K5 overexpressing and its vector control clones. β-actin was used as the loading control in the western blotting experiment. (C) Graph shows quantitation of western blot using densitometry. Fold-change in K5, K14 and involucrin protein level in K5 overexpressing clone is shown relative to that of its vector control clone. Error bars denote ± SEM from three independent experiments.
(TIF)

S13 Fig K14 re-expression alone in vimentin knockdown background failed to rescue the protein level of differentiation specific marker involucrin. (A) Immunofluorescence (Bar: 10 μm) images shows K14 overexpression in overexpressing (K14) and its vector control clone (K14vc). K5 levels remained unchanged between the clones. (B) Western blot analysis shows protein level of K14, K5 and involucrin in K14 overexpressing and its vector control clones. β-actin was used as the loading control in the western blotting experiment. (C) Graph shows quantitation of western blot using densitometry. Fold-change in K14, K5 and involucrin protein level in K14 overexpressing clone is shown relative to that of its vector control clone. Error bars denote ± SEM from three independent experiments.
(TIF)

S14 Fig Tumors formed in mice from K5/K14 overexpressing cells showed a decrease in the expression of involucrin. Representative images (Bar: 200μm) of IHC staining for expression of involucrin and PCNA in tumor tissues of mice, injected with either K5/K14 overexpressing or its vector control clones. The negative control images represent tissue sections incubated with serum from non-immunized mice in place of primary antibodies.
(TIF)

S15 Fig Graphs showing fold change in the protein level of ΔNp63 and its related molecules. Graphs show quantitation of western blots using densitometry. (A) Fold-change in ΔNp63, p21 and p27 protein level in vimentin knockdown clones is shown relative to that of its vector control clone. (B) Fold-change in flag- tagged ΔNp63α protein level in flag-ΔNp63α overexpressing clone is shown relative to that of its vector control clone. (C) Fold-change in vimentin protein level in vimentin knockdown (shvim2), flag-ΔNp63α overexpressing (ΔNp63α) and its vector control clone (vbb) is shown relative to that of vector control clone (pTU6vc). (D) Fold-change in K5 and K14 protein level in flag-ΔNp63α overexpressing clone is shown relative to that of its vector control clone. Error bars denote ± SEM from three independent experiments.
(TIF)

S16 Fig Confocal images showing filament organization of K5 and K14, upon re-expression of ΔNp63α in vimentin knockdown cells. (A and B) Confocal microscopy analysis (Bar: 10μm) shows levels and filament networks of K5 and K14 respectively in ΔNp63α (flag tagged ΔNp63α overexpressing) as compared to vbb (vector control clone).
(TIF)

S17 Fig Oct-4 mRNA levels did not change significantly upon ΔNp63α re-expression. qRT-PCR analysis of Oct-4 in flag-ΔNp63α overexpressing and its vector control clones.
(TIF)

S18 Fig Notch1 (independently or through NF-κB) may regulate the expression of ΔNp63. (A) Western blot analysis shows the protein levels of notch intracellular domain (NICD) in vimentin knockdown and its vector control cells. (B) The confocal images (Bar: 20μm) show the distribution of NF-κB (p65) (red) in the cytoplasmic vs. nuclear compartment in vimentin knockdown and its vector control cells. The nuclei (blue) were stained with DAPI. (C) Subcellular fractionation was carried out to separate cytoplasmic and nuclear fractions of vimentin knockdown and vector control clones. Western blot analysis shows the distribution of p65 in cytoplasmic and nuclear fractions. (D) Western blot analysis shows the protein levels of notch intracellular domain (NICD) in flag-ΔNp63α and its vector control clones. (E) The confocal images (Bar: 20μm) show the distribution of NF-κB (p65) (red) in the cytoplasmic vs. nuclear compartment in flag-ΔNp63α and its vector control clones. The nuclei (blue) were stained with DAPI. (F) Subcellular fractionation was carried out to separate cytoplasmic and nuclear fractions of flag-ΔNp63α and its vector control clones. Western blot analysis shows the distribution of p65 in cytoplasmic and nuclear fractions. β-actin was used as a loading control for the whole cell lysates. α-tubulin was used as a loading control for the cytoplasmic fraction while histone H3 protein was used as a loading control for the nuclear fraction. All the experiments were repeated independently in triplicates.
(TIF)

S19 Fig Graphs showing fold change in the protein level of NICD, nuclear NF-κB and transcript levels of their respective target genes. (A) Fold-change in NICD protein level in vimentin knockdown clone is shown relative to that of its vector control clone. Also, fold-change in NICD protein level in flag-ΔNp63α overexpressing clone is shown relative to that of its vector control clone. (B) Fold-change in NF-κB (p65) nuclear protein level in vimentin knockdown clone is shown relative to that of its vector control clone. Also, fold-change in NF-κB (p65) nuclear protein level in flag-ΔNp63α overexpressing clone is shown relative to that of its vector control clone. Fold change for nuclear levels of NF-κB was calculated by normalizing to its respective histone H3 nuclear levels. (C) qRT-PCR analysis of Hes1 in vimentin knockdown clone is shown relative to that of its vector control clone. Also, qRT-PCR analysis of Hes1 in flag-ΔNp63α overexpressing clone is shown relative to that of its vector control clone. (D) qRT-PCR analysis of IκBα in vimentin knockdown clone is shown relative to that of its vector control clone. Also, qRT-PCR analysis of IκBα in flag-ΔNp63α overexpressing clone is shown relative to that of its vector control clone. Error bars denote ± SEM from three independent experiments.
(TIF)

S20 Fig Immunohistochemistry analysis on human oral tumor tissues showed positive correlation between K5/K14 and vimentin expression. (A) The upper panel show images (Bar: 200μm) of IHC staining for K1, K5, K14 and vimentin expression in cut margin tissues while lower panel show images (Bar: 200μm) of IHC staining for K1, K5, K14 and vimentin expression in tumor tissues. The negative control images represent tissue sections incubated with serum from non-immunized mice in place of primary antibodies. (B) Kaplan-Meier survival analysis (n = 91) of high vimentin-K5-K14-low K1 expression vs. the other combinations of vimentin-K5-K14-K1 expression.
(TIF)

S1 Table List of cell lines used in this study. The table shows a list of cell lines along with their particulars.
(TIF)

S2 Table Antibodies used in this study. The table shows a list of antibodies along with their particulars.
(TIF)

S3 Table Reagents used in this study. The table shows a list of reagents along with their particulars.
(TIF)

S4 Table Primers used in this study. The table shows a list of primer sequences used for RT-PCR and qRT-PCR analysis.
(TIF)

S5 Table Correlations of vimentin-K14 expression with clinicopathological parameters of the oral cancer patients (n = 100). (TIF)

S6 Table Correlations of high vimentin-K5-K14-low K1 expression with clinicopathological parameters of the oral cancer patients (n = 100). (TIF)
