# Supplementary figures and images for: Vimentin regulates differentiation switch via modulation of keratin 14 levels and their expression together correlates with poor prognosis in oral cancer patients
Source: PLoS One. 2017 Feb 22;12(2):e0172559. doi: 10.1371/journal.pone.0172559 (PMC5321444; doi:10.1371/journal.pone.0172559)

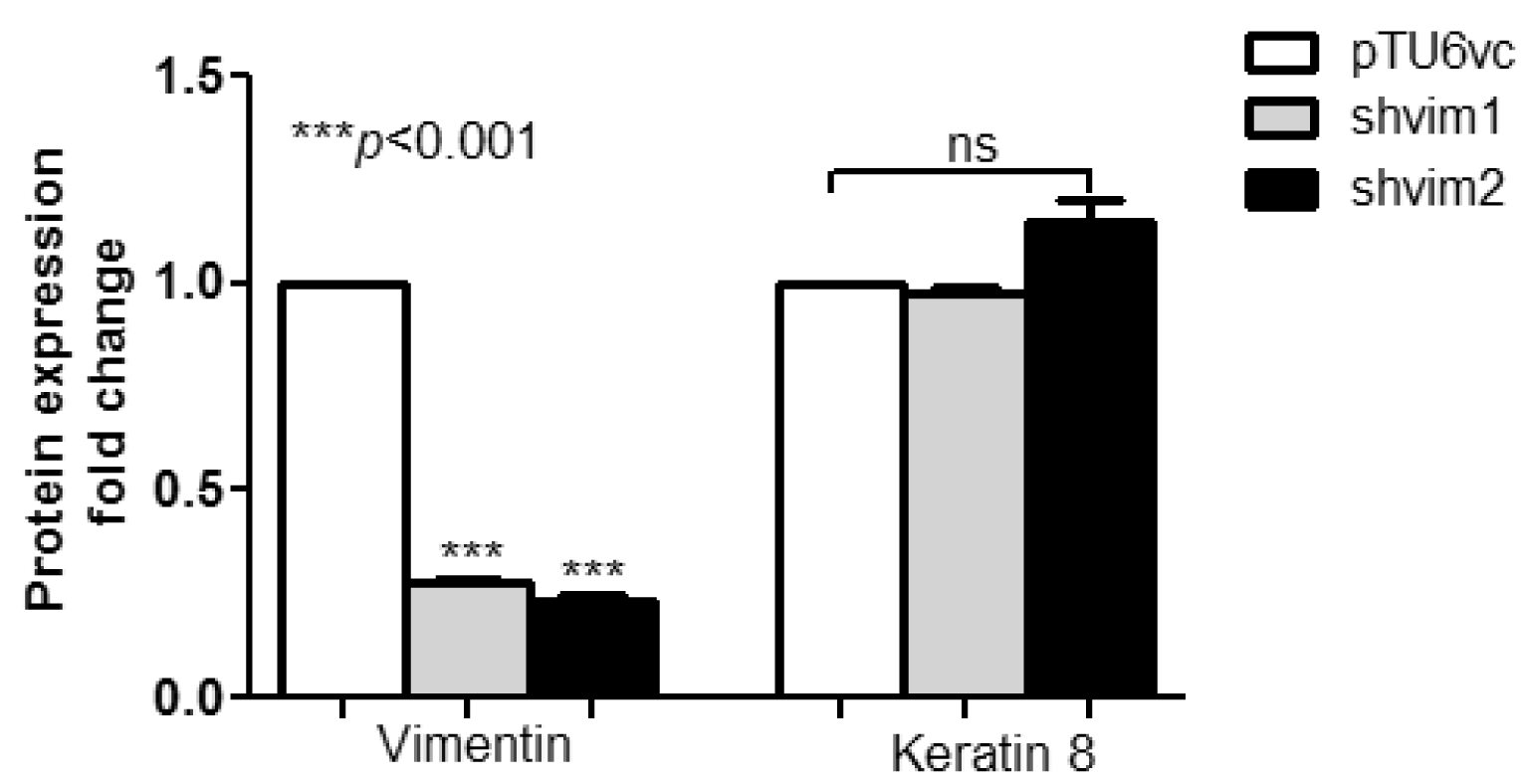

Supplement: S1 Fig — Graph shows quantitation of western blots using densitometry. Fold-change in vimentin and K8 protein level in vimentin knockdown clones is shown relative to that of its vector control clone. Error bars denote ± SEM. from three independent experiments. (TIF) [file pone.0172559.s001.tif]

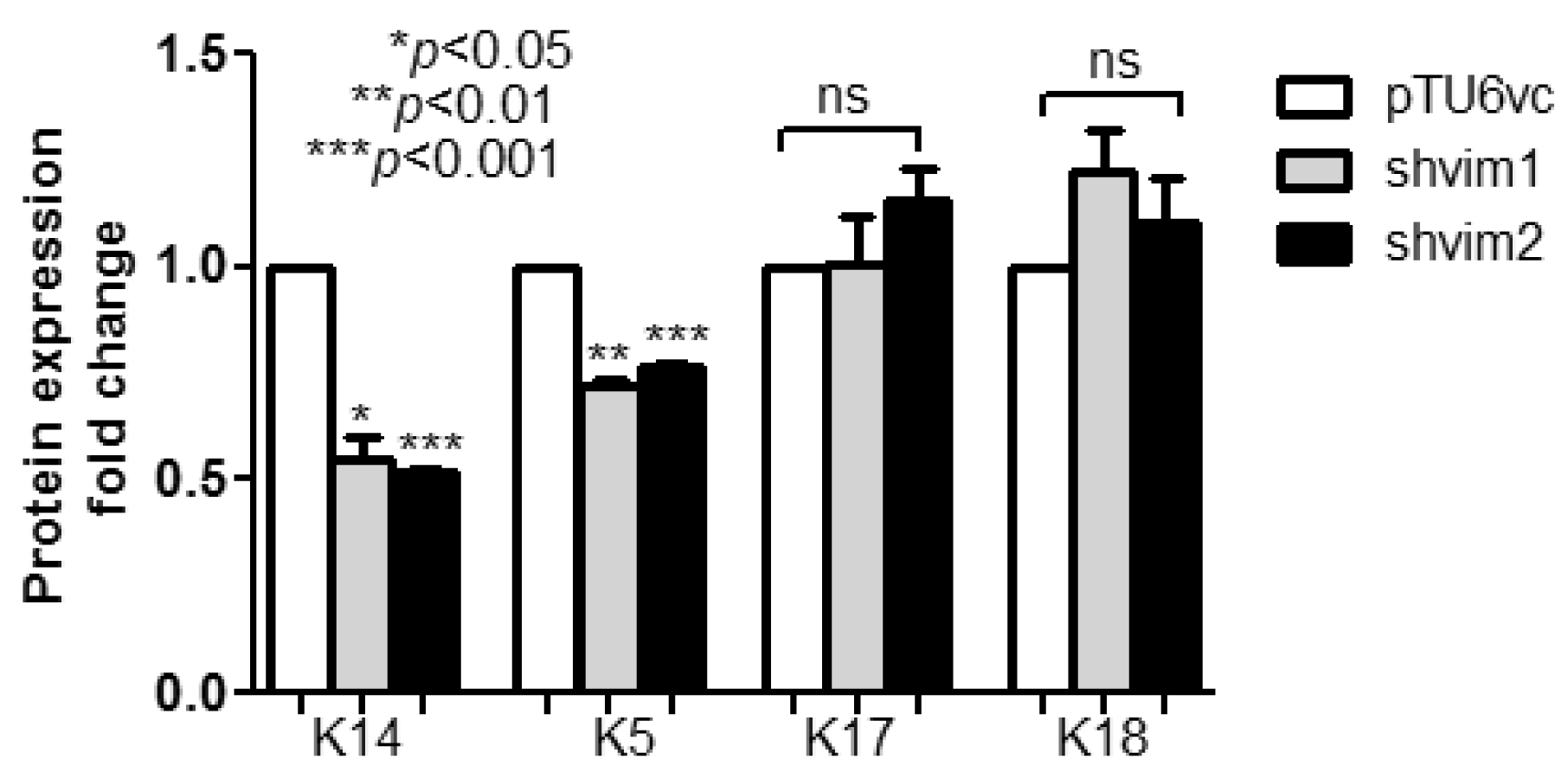

Supplement: S2 Fig — Graph shows quantitation of western blots using densitometry. Fold-change in K14, K5, K17 and K18 protein level in vimentin knockdown clones is shown relative to that of its vector control clone. Error bars denote ±S.E.M. from three independent experiments. (TIF) [file pone.0172559.s002.tif]

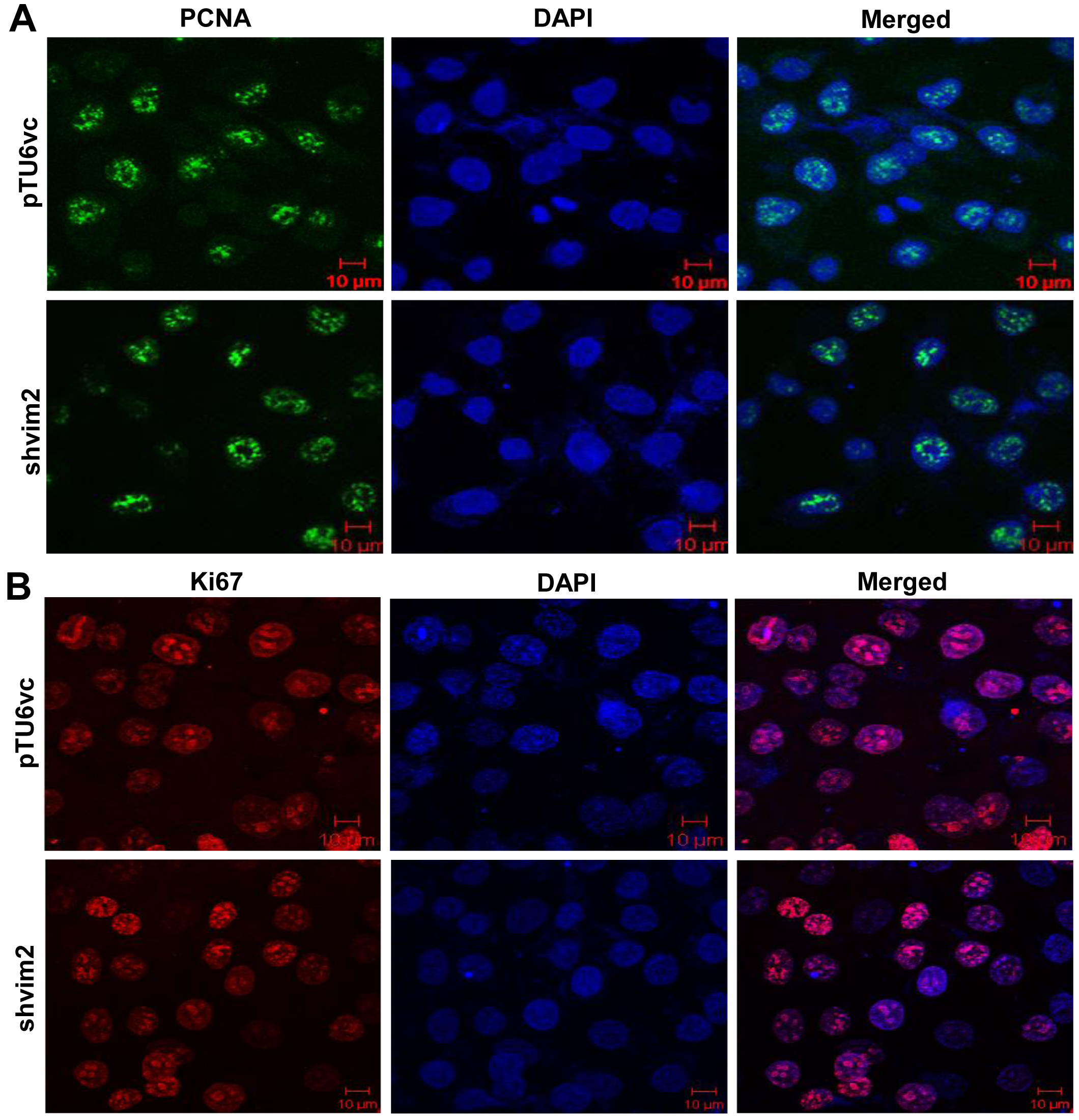

Supplement: S3 Fig — (A) Representative immunofluorescence images (Bar: 10μm) of PCNA (green) staining in vimentin knockdown and vector control clones. (B) Representative immunofluorescence images (Bar: 10μm) of Ki67 (red) staining in vimentin knockdown and vector control clones. (TIF) [file pone.0172559.s003.tif]

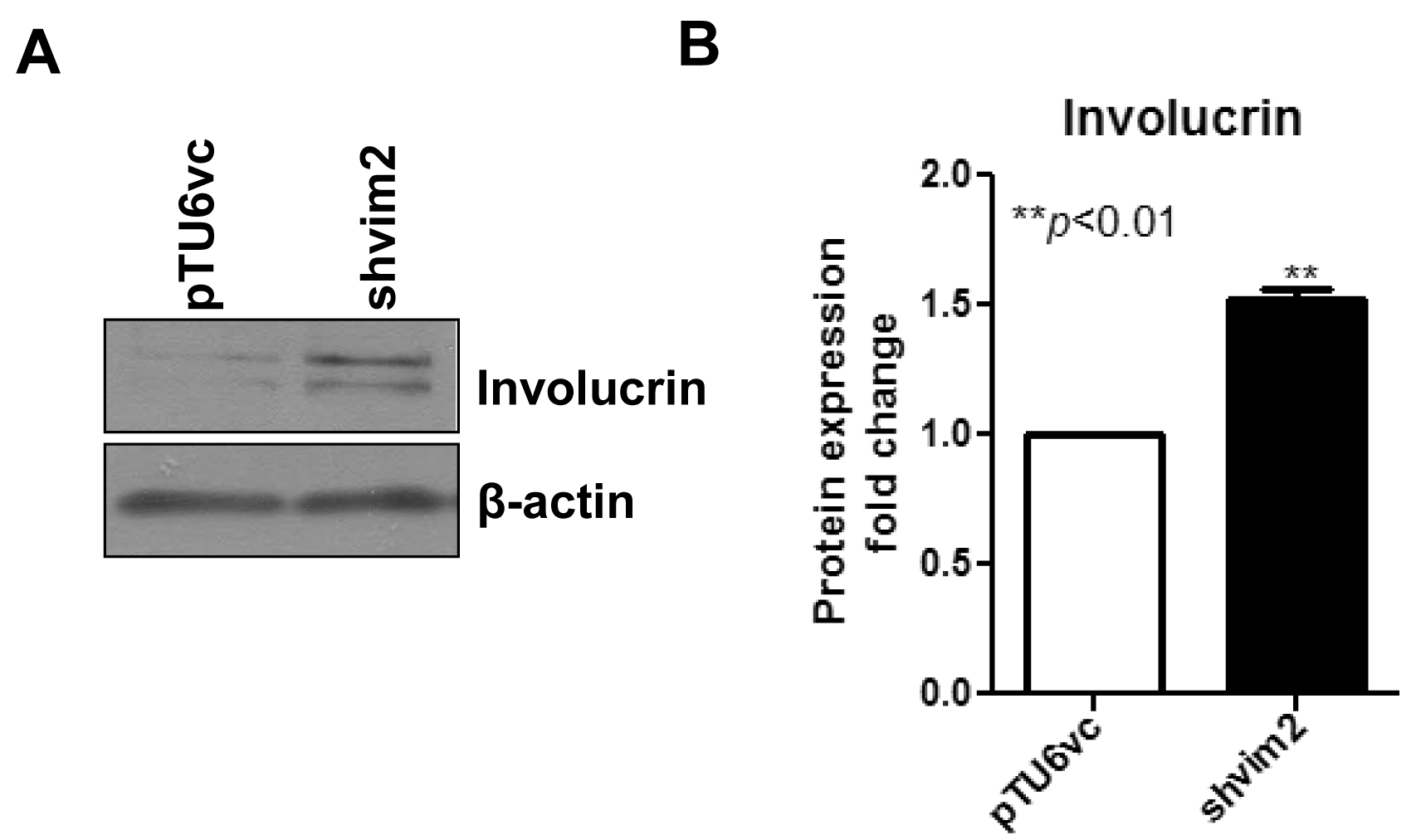

Supplement: S4 Fig — (A) Western blot analysis shows protein level of involucrin from whole cell lysates of vimentin knockdown and its vector control clones. (B) Graph shows quantitation of western blot using densitometry. Fold-change in involucrin protein level in vimentin knockdown clone is shown relative to that of its vector control clone. Error bars denote ± SEM. from three independent experiments. (TIF) [file pone.0172559.s004.tif]

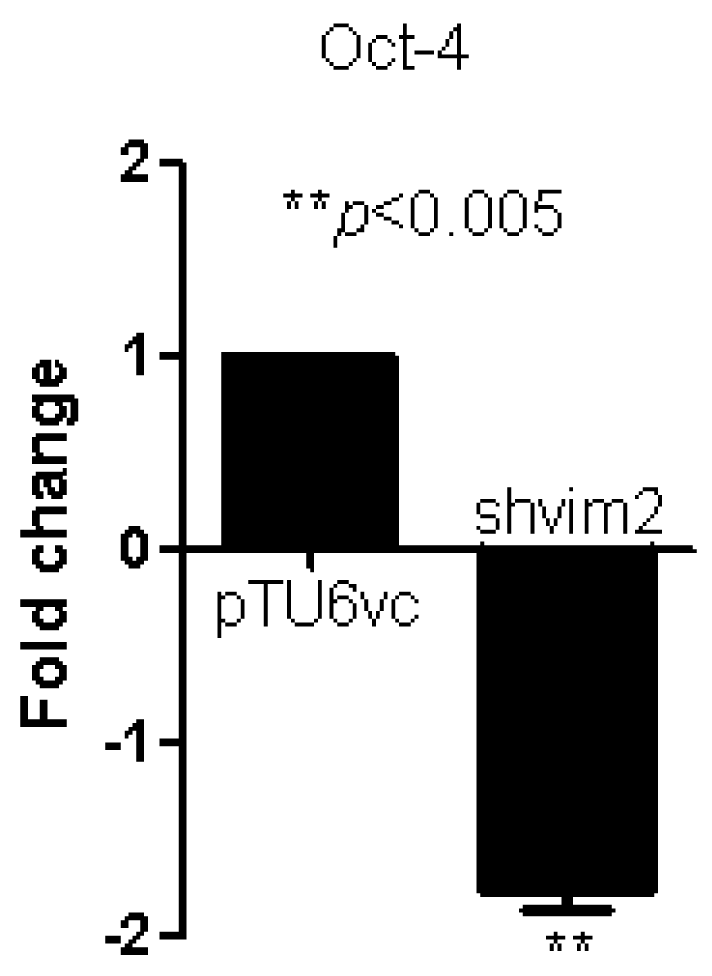

Supplement: S5 Fig — QRT-PCR analysis of Oct-4 in vimentin knockdown and its vector control clones. (TIF) [file pone.0172559.s005.tif]

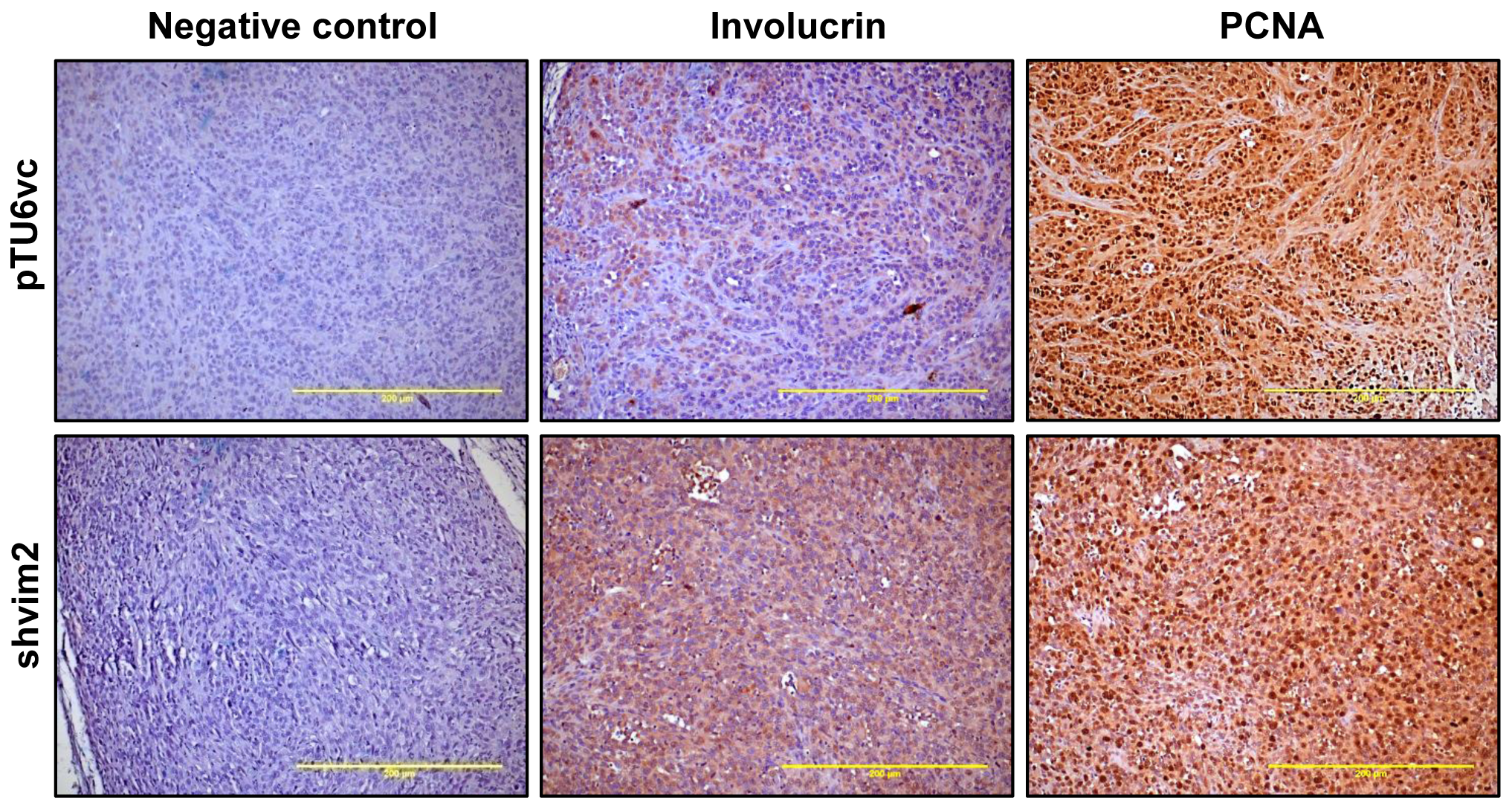

Supplement: S6 Fig — Representative images (Bar: 200μm) of IHC staining for expression of involucrin and PCNA in tumor tissues of mice, injected with either vimentin knockdown or vector control clones. The negative control images represent tissue sections incubated with serum from non-immunized mice in place of primary antibodies. (TIF) [file pone.0172559.s006.tif]

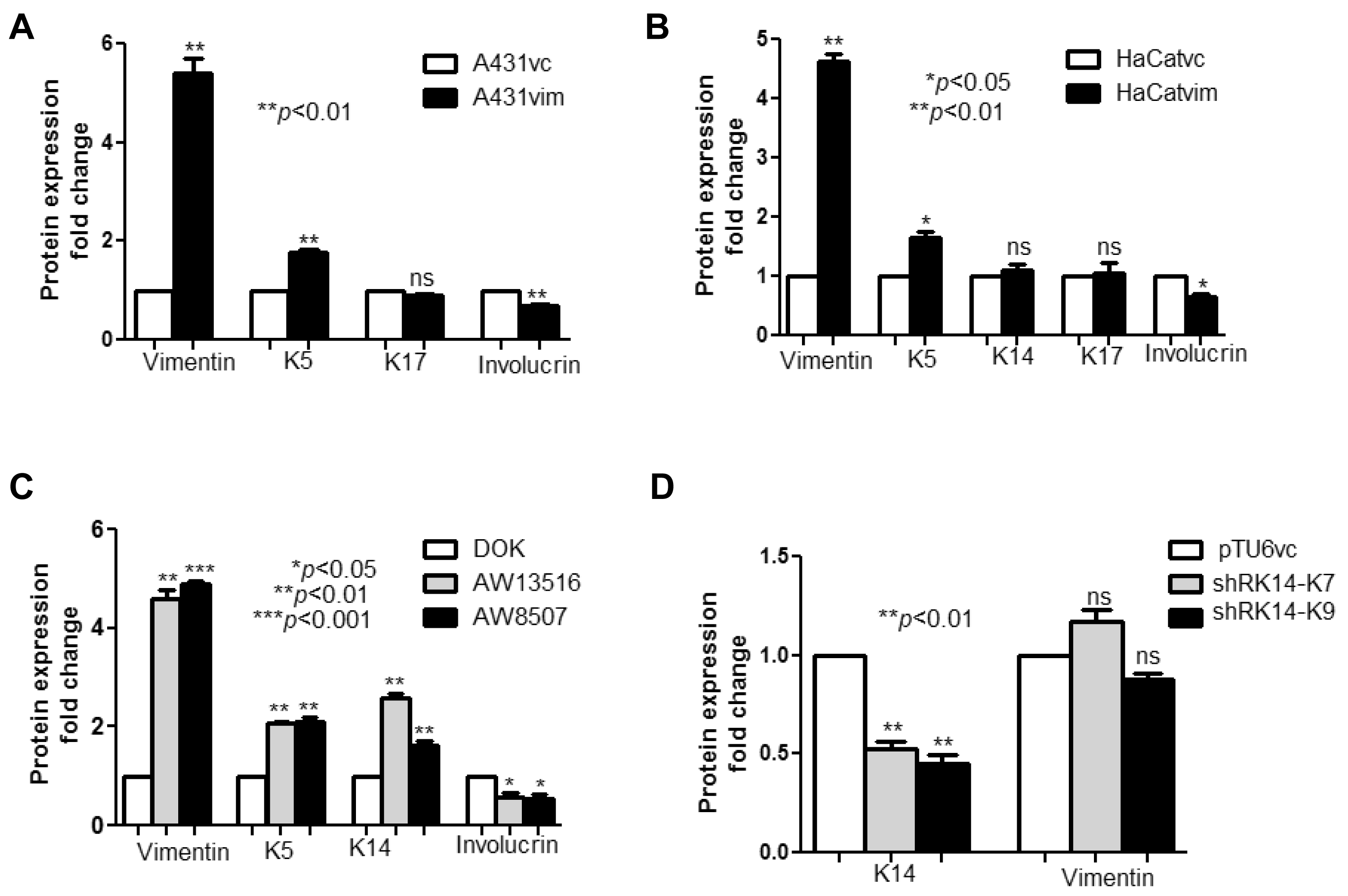

Supplement: S7 Fig — Graphs show quantitation of western blots using densitometry. (A and B) Fold-change in vimentin, K5, K14, K17 and involucrin protein level in vimentin overexpressing clones of A431vim and HaCatvim is shown relative to its respective vector control clones A431vc and HaCatvc. (C) Fold-change in vimentin, K5, K14, K17 and involucrin protein level in AW13516 and AW8507 cells is shown relative to that of DOK cells. (D) Fold-change in K14 and vimentin protein level in K14 knockdown clones is shown relative to its respective vector control clone. Error bars denote ± SEM from three independent experiments. (TIF) [file pone.0172559.s007.tif]

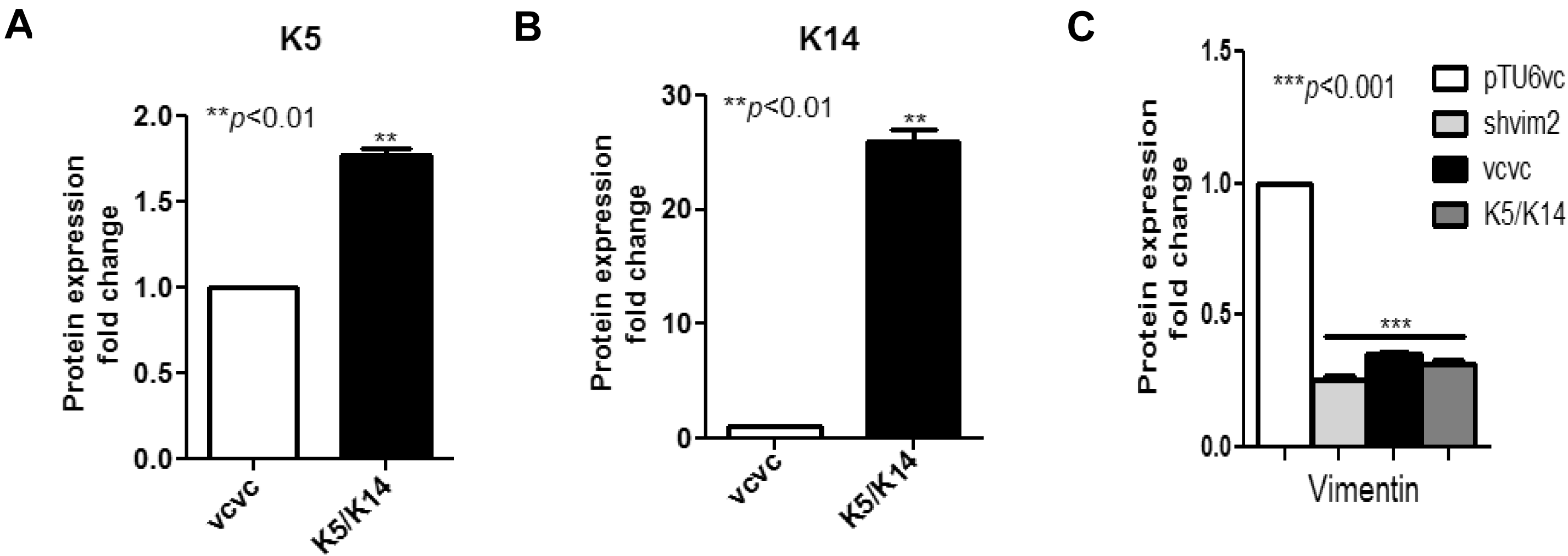

Supplement: S8 Fig — Graphs show quantitation of western blots using densitometry. (A and B) Fold-change in K5 and K14 protein level in K5/K14 overexpressing clone is shown relative to that of its vector control clone. (C) Fold-change in vimentin protein level in vimentin knockdown (shvim2), K5/K14 overexpressing clone (K5/K14) and its vector control clone (vcvc) is shown relative to that of vector control clone (pTU6vc). Error bars denote ± SEM from three independent experiments. (TIF) [file pone.0172559.s008.tif]

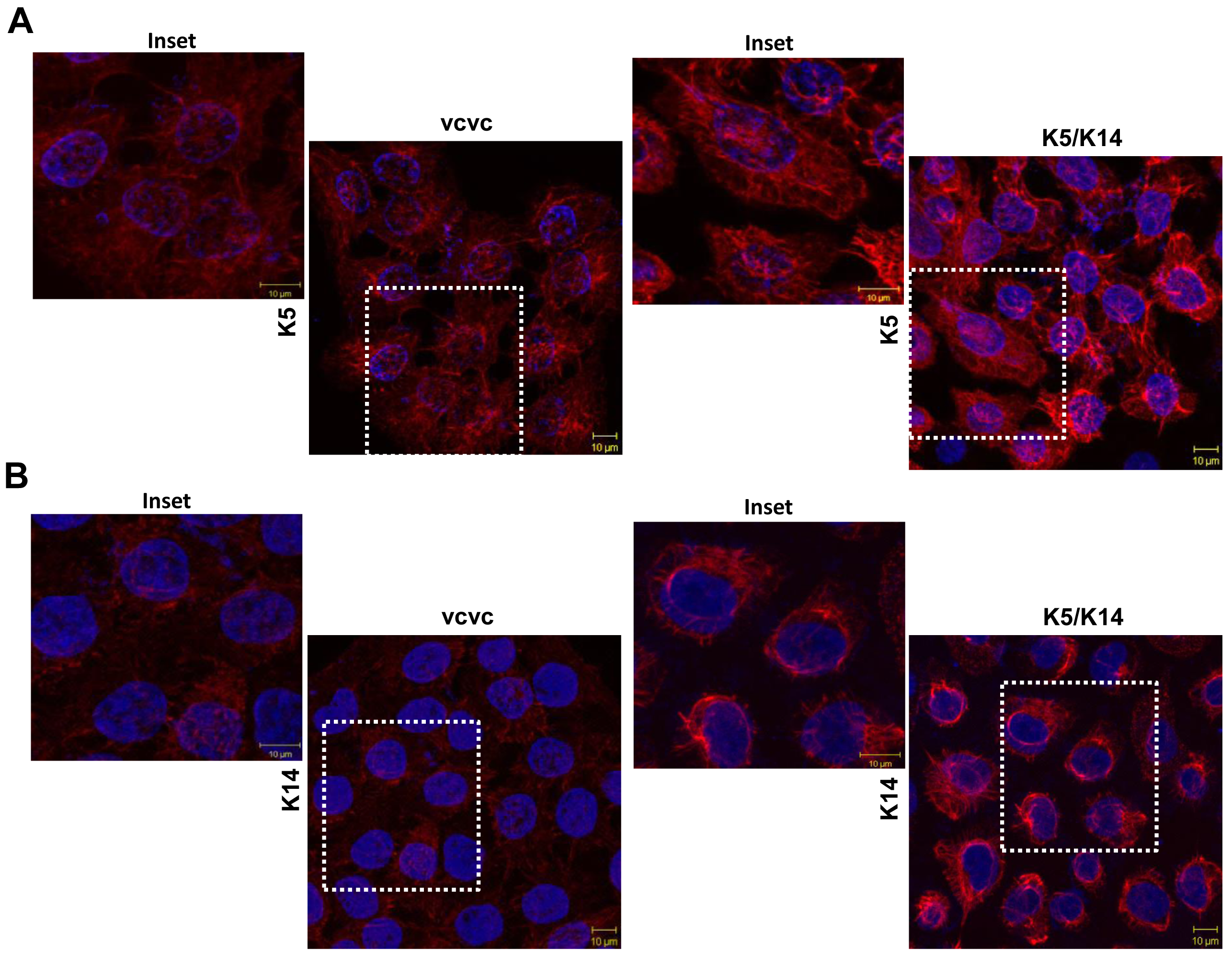

Supplement: S9 Fig — (A and B) Confocal microscopy analysis (Bar: 10μm) shows levels and filament networks of K5 and K14 respectively in K5/K14 (K5 and K14 overexpressing) as compared to its vector control vcvc clones (empty vectors of K5 and K14 together). (TIF) [file pone.0172559.s009.tif]

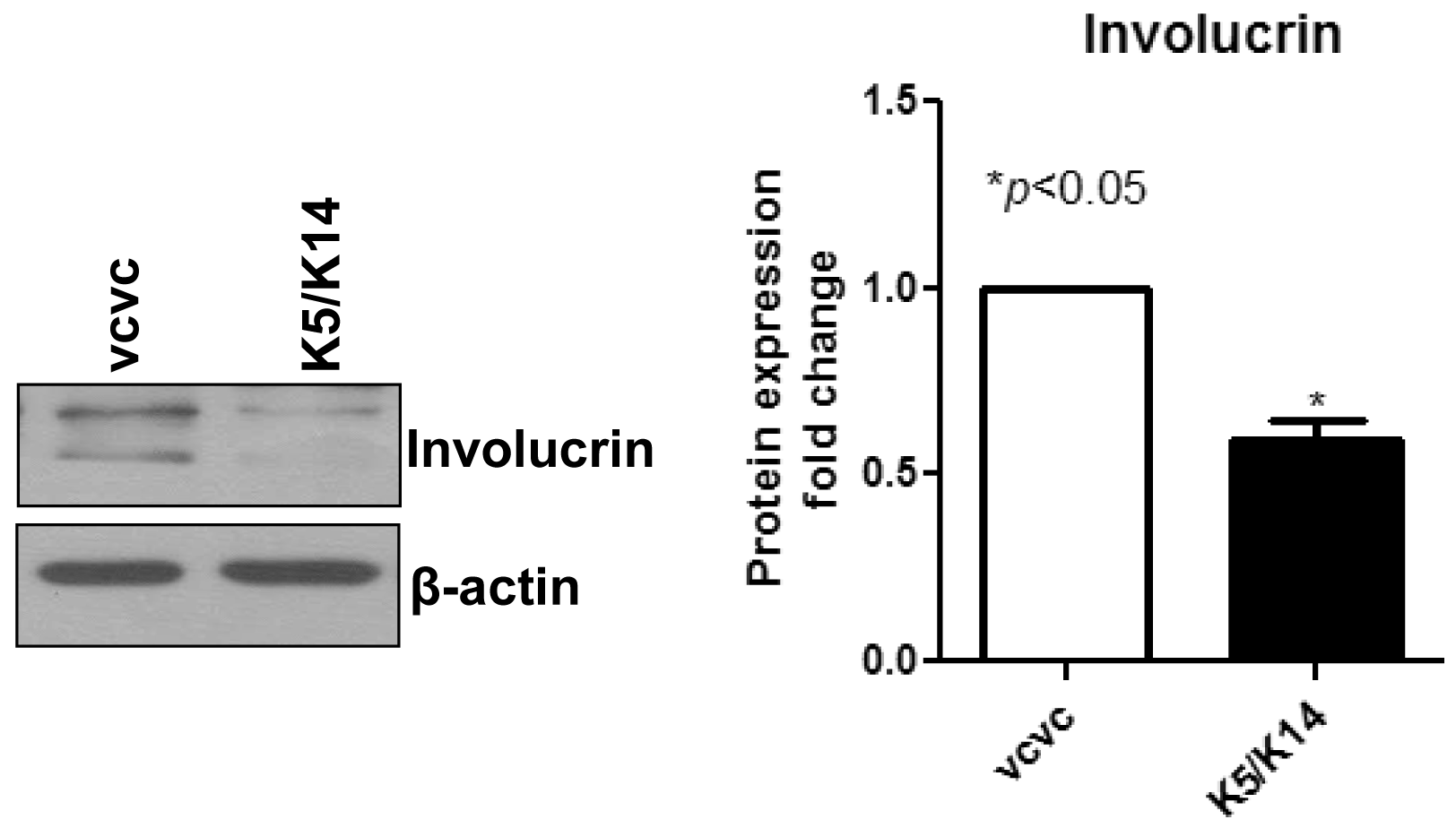

Supplement: S10 Fig — (A) Western blot analysis shows protein level of involucrin from whole cell lysates of K5/K14 overexpressing as compared to its vector control clones. (B) Graph shows quantitation of western blot using densitometry. Fold-change in involucrin protein level in K5/K14 overexpressing clone is shown relative to that of its vector control clone. Error bars denote ± SEM from three independent experiments. (TIF) [file pone.0172559.s010.tif]

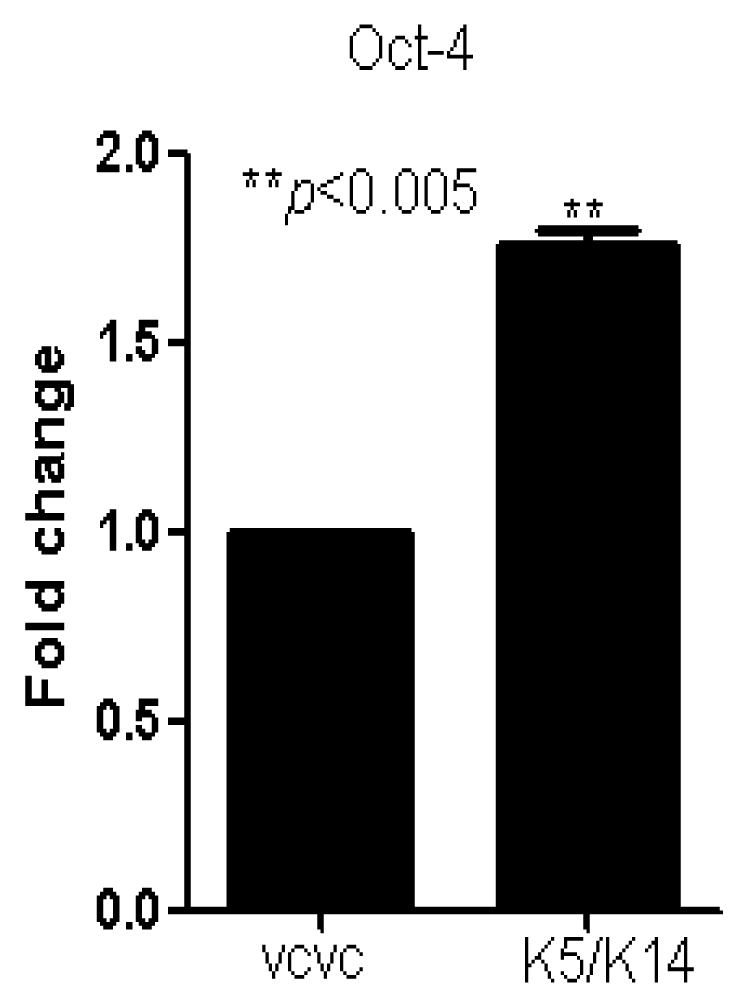

Supplement: S11 Fig — qRT-PCR analysis of Oct-4 in K5/K14 overexpressing and its vector control clones. (TIF) [file pone.0172559.s011.tif]

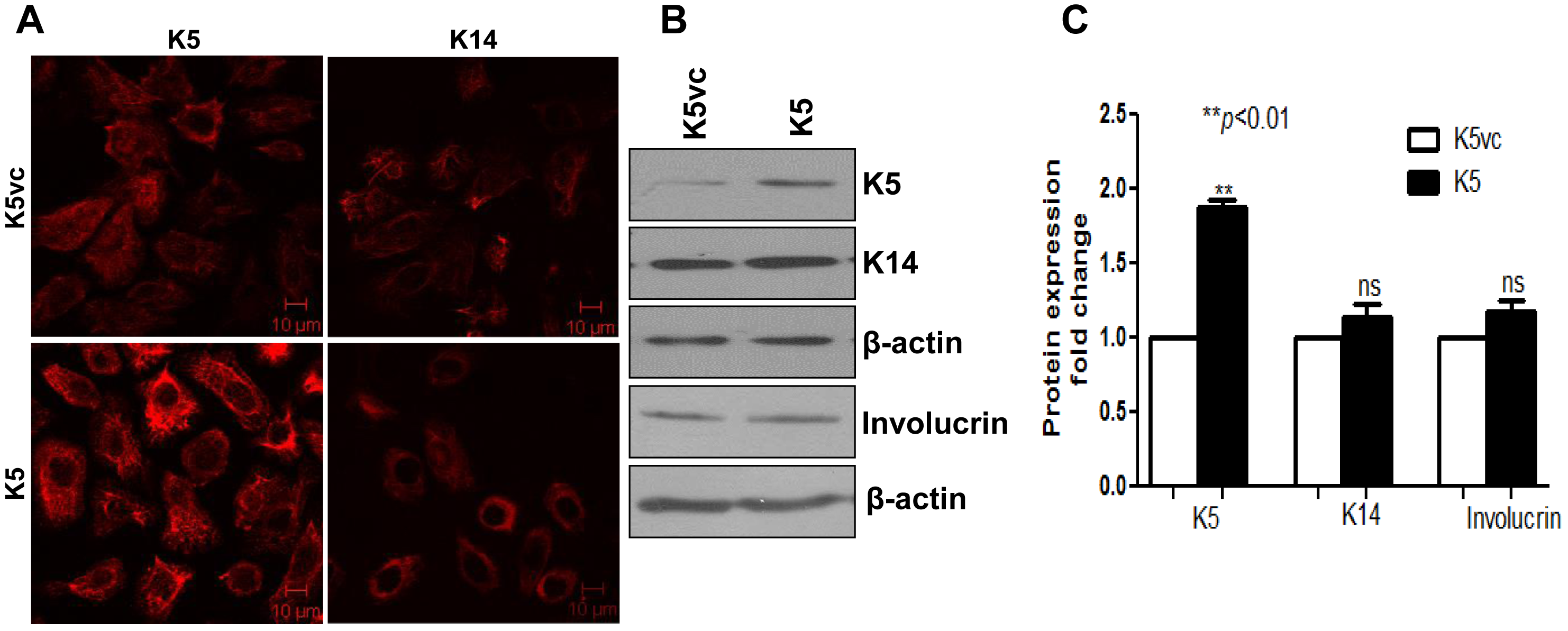

Supplement: S12 Fig — (A) Immunofluorescence (Bar: 10 μm) images of K5 overexpressing (K5) and its vector control clone (K5vc) using antibodies against K5 and K14. (B) Western blot analysis shows protein level of K5, K14 and involucrin in K5 overexpressing and its vector control clones. β-actin was used as the loading control in the western blotting experiment. (C) Graph shows quantitation of western blot using densitometry. Fold-change in K5, K14 and involucrin protein level in K5 overexpressing clone is shown relative to that of its vector control clone. Error bars denote ± SEM from three independent experiments. (TIF) [file pone.0172559.s012.tif]

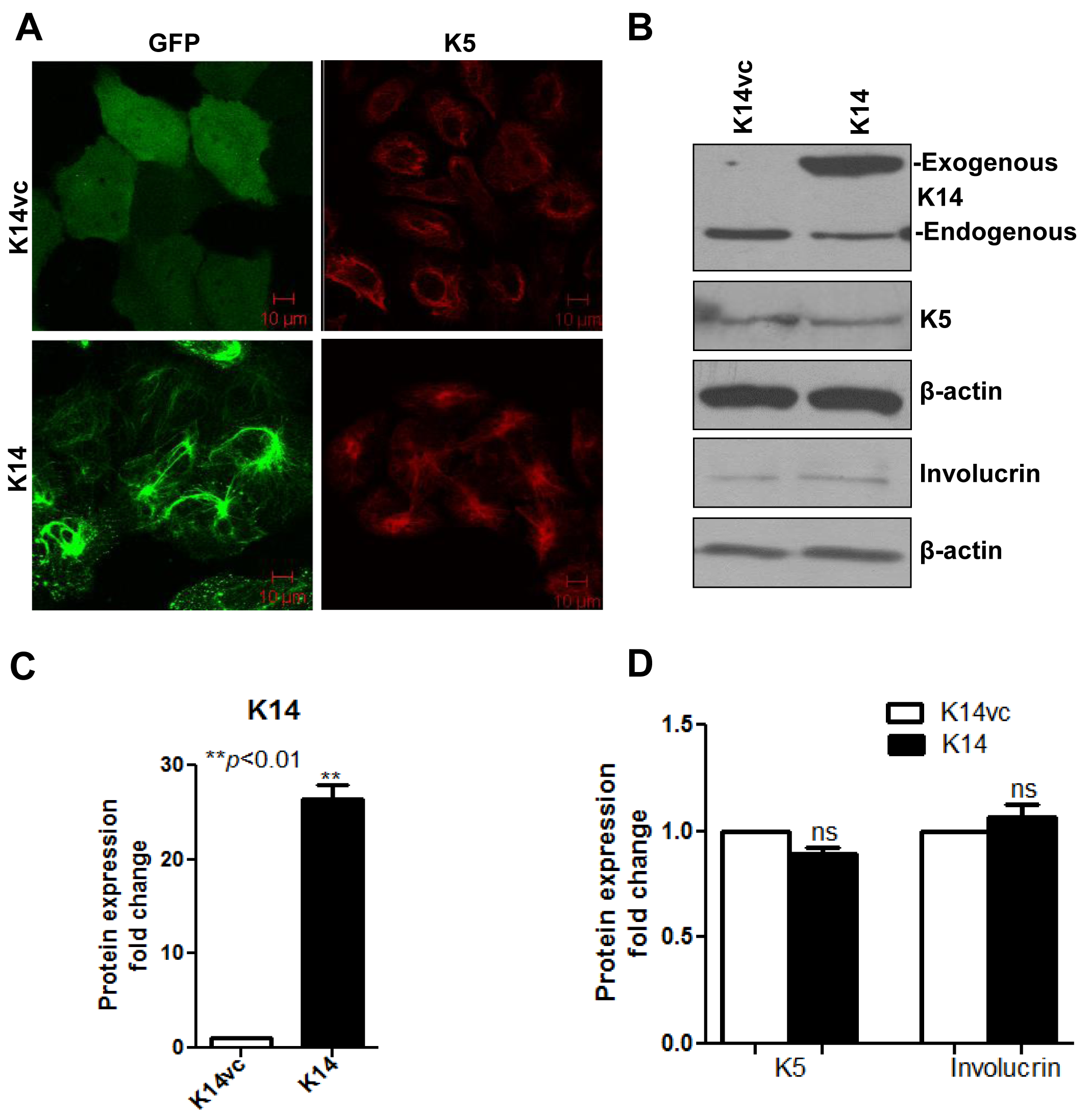

Supplement: S13 Fig — (A) Immunofluorescence (Bar: 10 μm) images shows K14 overexpression in overexpressing (K14) and its vector control clone (K14vc). K5 levels remained unchanged between the clones. (B) Western blot analysis shows protein level of K14, K5 and involucrin in K14 overexpressing and its vector control clones. β-actin was used as the loading control in the western blotting experiment. (C) Graph shows quantitation of western blot using densitometry. Fold-change in K14, K5 and involucrin protein level in K14 overexpressing clone is shown relative to that of its vector control clone. Error bars denote ± SEM from three independent experiments. (TIF) [file pone.0172559.s013.tif]

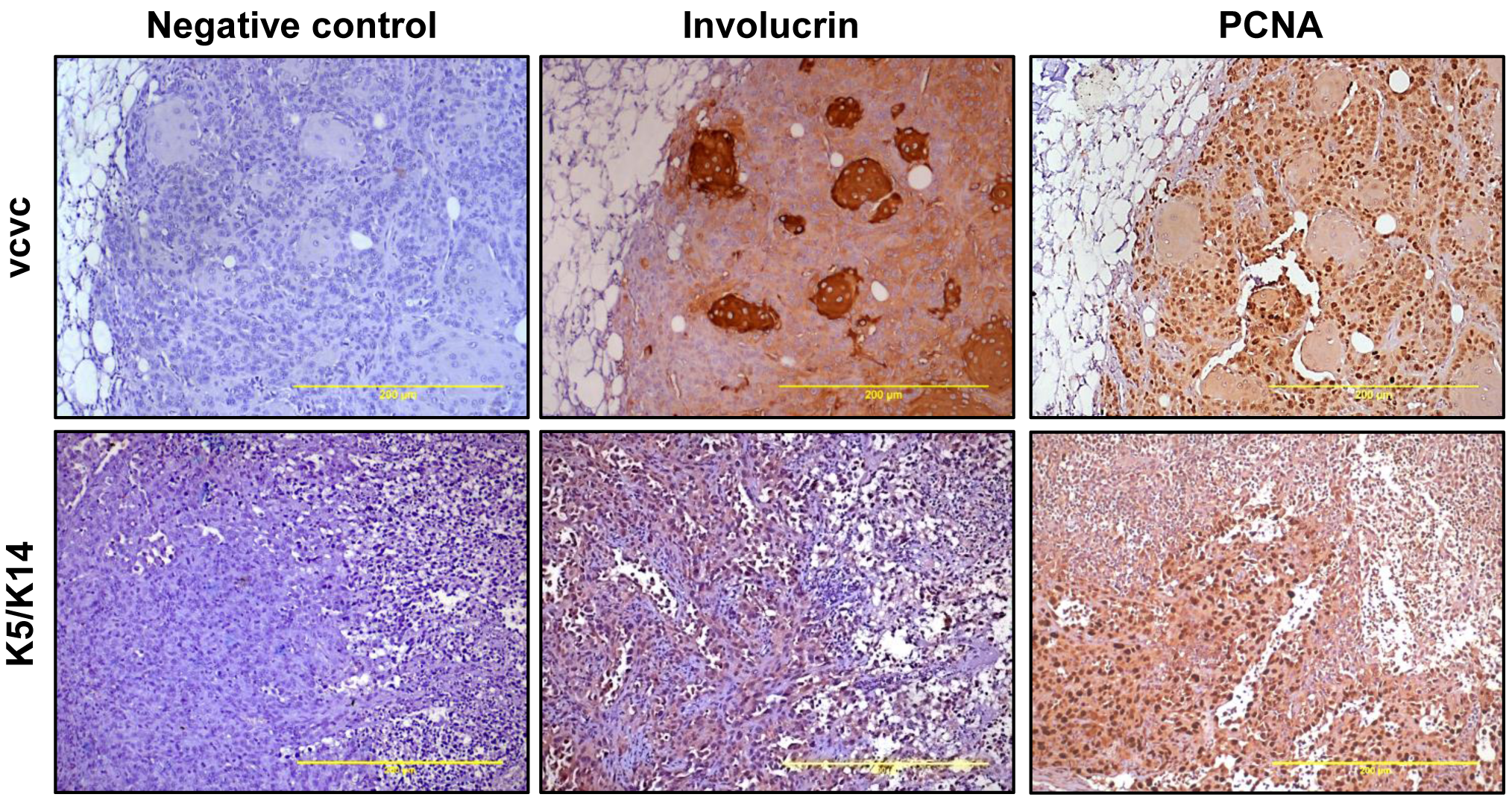

Supplement: S14 Fig — Representative images (Bar: 200μm) of IHC staining for expression of involucrin and PCNA in tumor tissues of mice, injected with either K5/K14 overexpressing or its vector control clones. The negative control images represent tissue sections incubated with serum from non-immunized mice in place of primary antibodies. (TIF) [file pone.0172559.s014.tif]

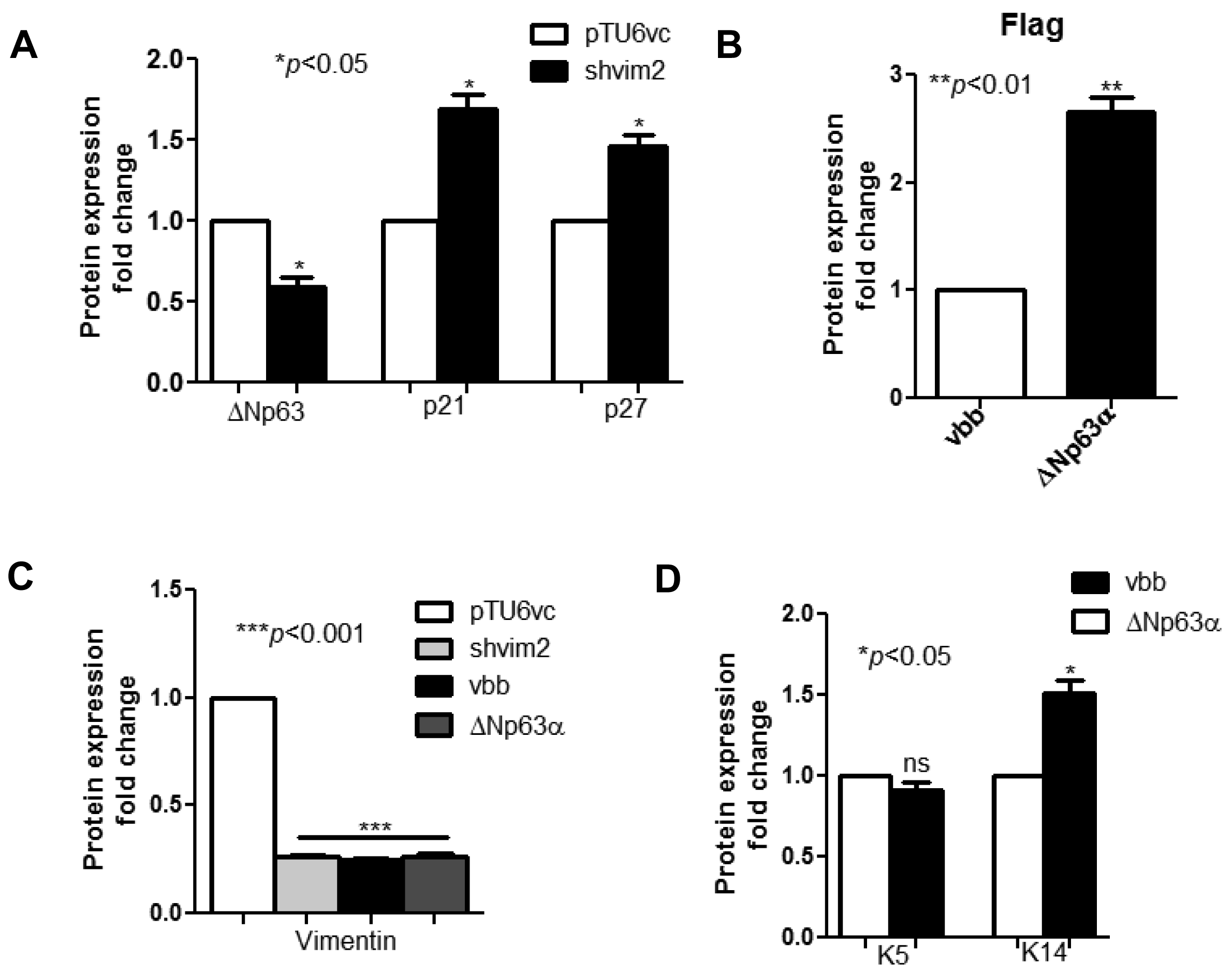

Supplement: S15 Fig — Graphs show quantitation of western blots using densitometry. (A) Fold-change in ΔNp63, p21 and p27 protein level in vimentin knockdown clones is shown relative to that of its vector control clone. (B) Fold-change in flag- tagged ΔNp63α protein level in flag-ΔNp63α overexpressing clone is shown relative to that of its vector control clone. (C) Fold-change in vimentin protein level in vimentin knockdown (shvim2), flag-ΔNp63α overexpressing (ΔNp63α) and its vector control clone (vbb) is shown relative to that of vector control clone (pTU6vc). (D) Fold-change in K5 and K14 protein level in flag-ΔNp63α overexpressing clone is shown relative to that of its vector control clone. Error bars denote ± SEM from three independent experiments. (TIF) [file pone.0172559.s015.tif]

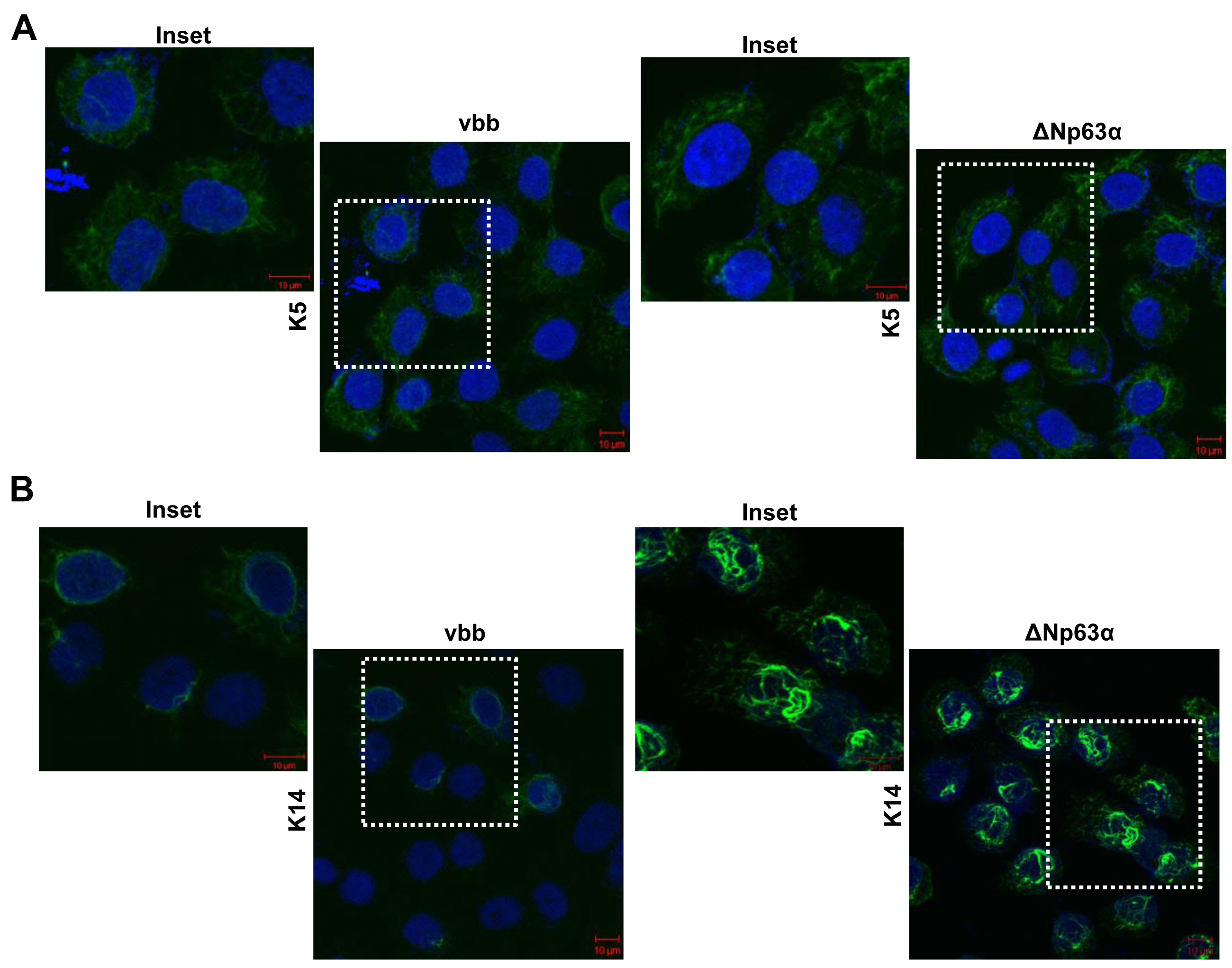

Supplement: S16 Fig — (A and B) Confocal microscopy analysis (Bar: 10μm) shows levels and filament networks of K5 and K14 respectively in ΔNp63α (flag tagged ΔNp63α overexpressing) as compared to vbb (vector control clone). (TIF) [file pone.0172559.s016.tif]

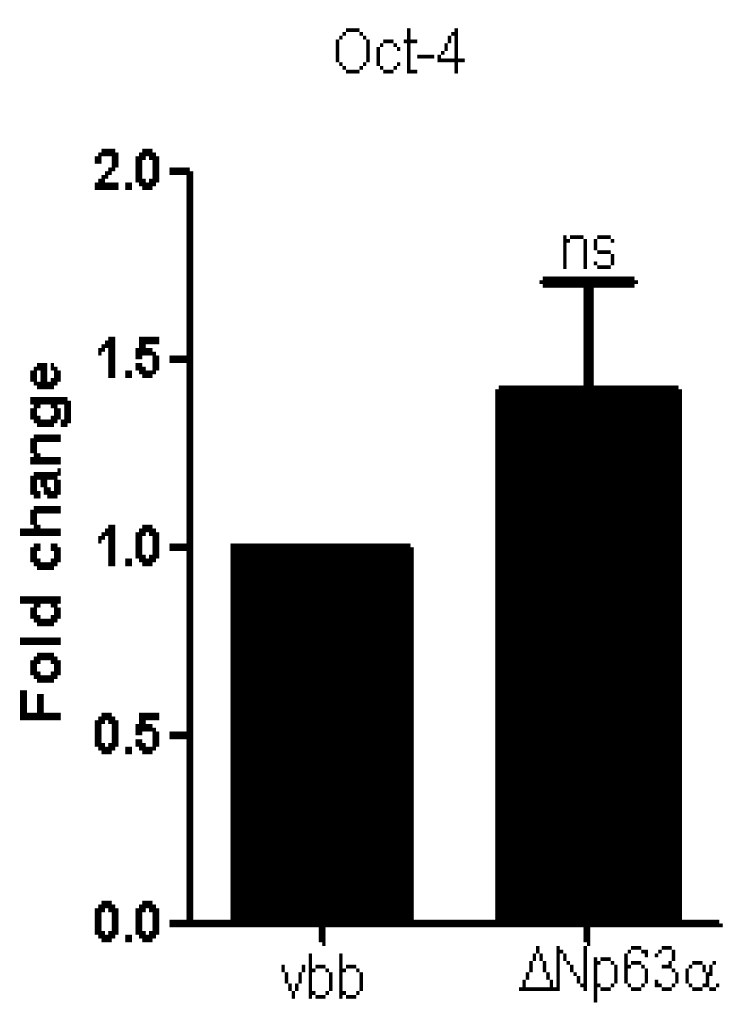

Supplement: S17 Fig — qRT-PCR analysis of Oct-4 in flag-ΔNp63α overexpressing and its vector control clones. (TIF) [file pone.0172559.s017.tif]

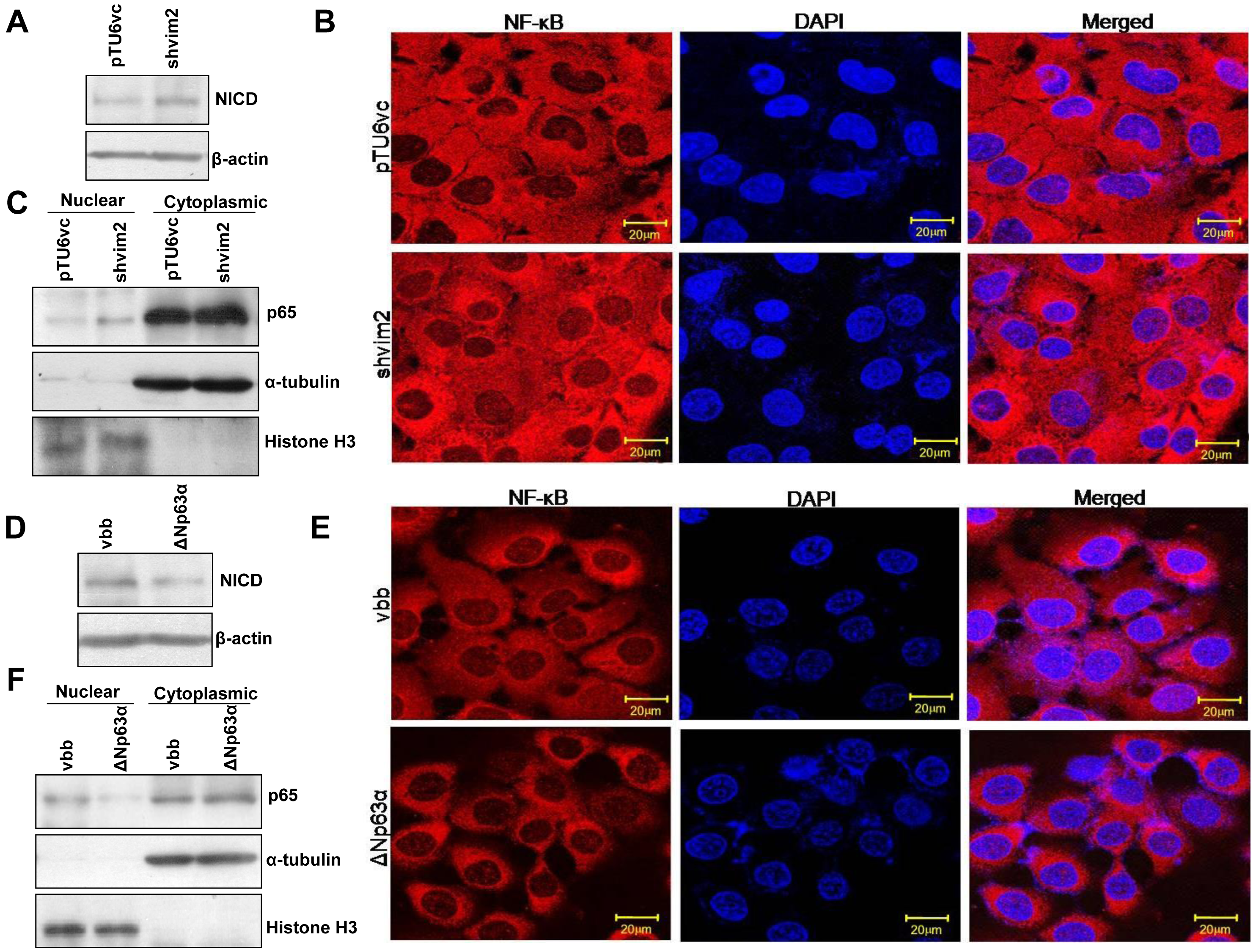

Supplement: S18 Fig — (A) Western blot analysis shows the protein levels of notch intracellular domain (NICD) in vimentin knockdown and its vector control cells. (B) The confocal images (Bar: 20μm) show the distribution of NF-κB (p65) (red) in the cytoplasmic vs. nuclear compartment in vimentin knockdown and its vector control cells. The nuclei (blue) were stained with DAPI. (C) Subcellular fractionation was carried out to separate cytoplasmic and nuclear fractions of vimentin knockdown and vector control clones. Western blot analysis shows the distribution of p65 in cytoplasmic and nuclear fractions. (D) Western blot analysis shows the protein levels of notch intracellular domain (NICD) in flag-ΔNp63α and its vector control clones. (E) The confocal images (Bar: 20μm) show the distribution of NF-κB (p65) (red) in the cytoplasmic vs. nuclear compartment in flag-ΔNp63α and its vector control clones. The nuclei (blue) were stained with DAPI. (F) Subcellular fractionation was carried out to separate cytoplasmic and nuclear fractions of flag-ΔNp63α and its vector control clones. Western blot analysis shows the distribution of p65 in cytoplasmic and nuclear fractions. β-actin was used as a loading control for the whole cell lysates. α-tubulin was used as a loading control for the cytoplasmic fraction while histone H3 protein was used as a loading control for the nuclear fraction. All the experiments were repeated independently in triplicates. (TIF) [file pone.0172559.s018.tif]

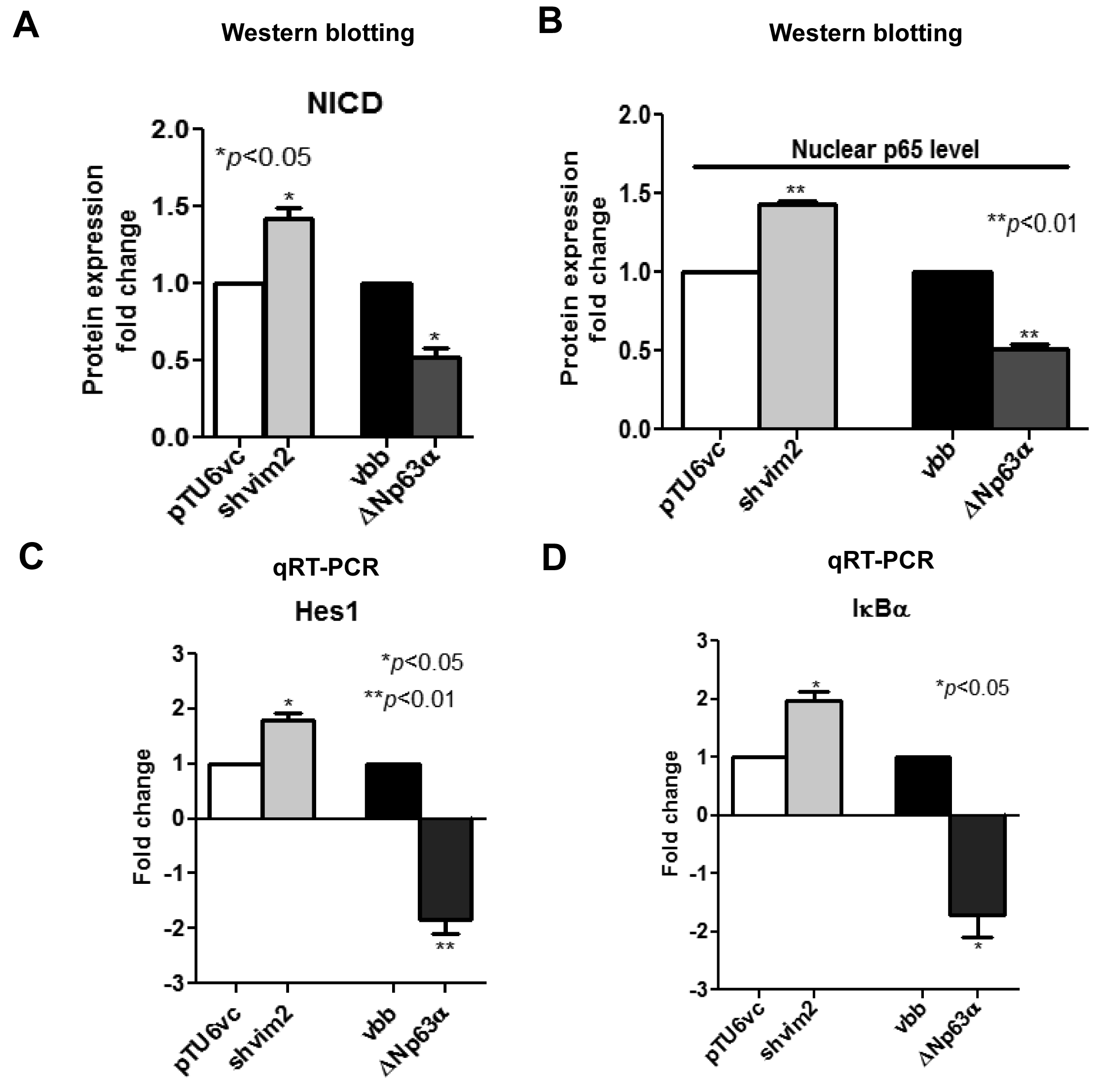

Supplement: S19 Fig — (A) Fold-change in NICD protein level in vimentin knockdown clone is shown relative to that of its vector control clone. Also, fold-change in NICD protein level in flag-ΔNp63α overexpressing clone is shown relative to that of its vector control clone. (B) Fold-change in NF-κB (p65) nuclear protein level in vimentin knockdown clone is shown relative to that of its vector control clone. Also, fold-change in NF-κB (p65) nuclear protein level in flag-ΔNp63α overexpressing clone is shown relative to that of its vector control clone. Fold change for nuclear levels of NF-κB was calculated by normalizing to its respective histone H3 nuclear levels. (C) qRT-PCR analysis of Hes1 in vimentin knockdown clone is shown relative to that of its vector control clone. Also, qRT-PCR analysis of Hes1 in flag-ΔNp63α overexpressing clone is shown relative to that of its vector control clone. (D) qRT-PCR analysis of IκBα in vimentin knockdown clone is shown relative to that of its vector control clone. Also, qRT-PCR analysis of IκBα in flag-ΔNp63α overexpressing clone is shown relative to that of its vector control clone. Error bars denote ± SEM from three independent experiments. (TIF) [file pone.0172559.s019.tif]

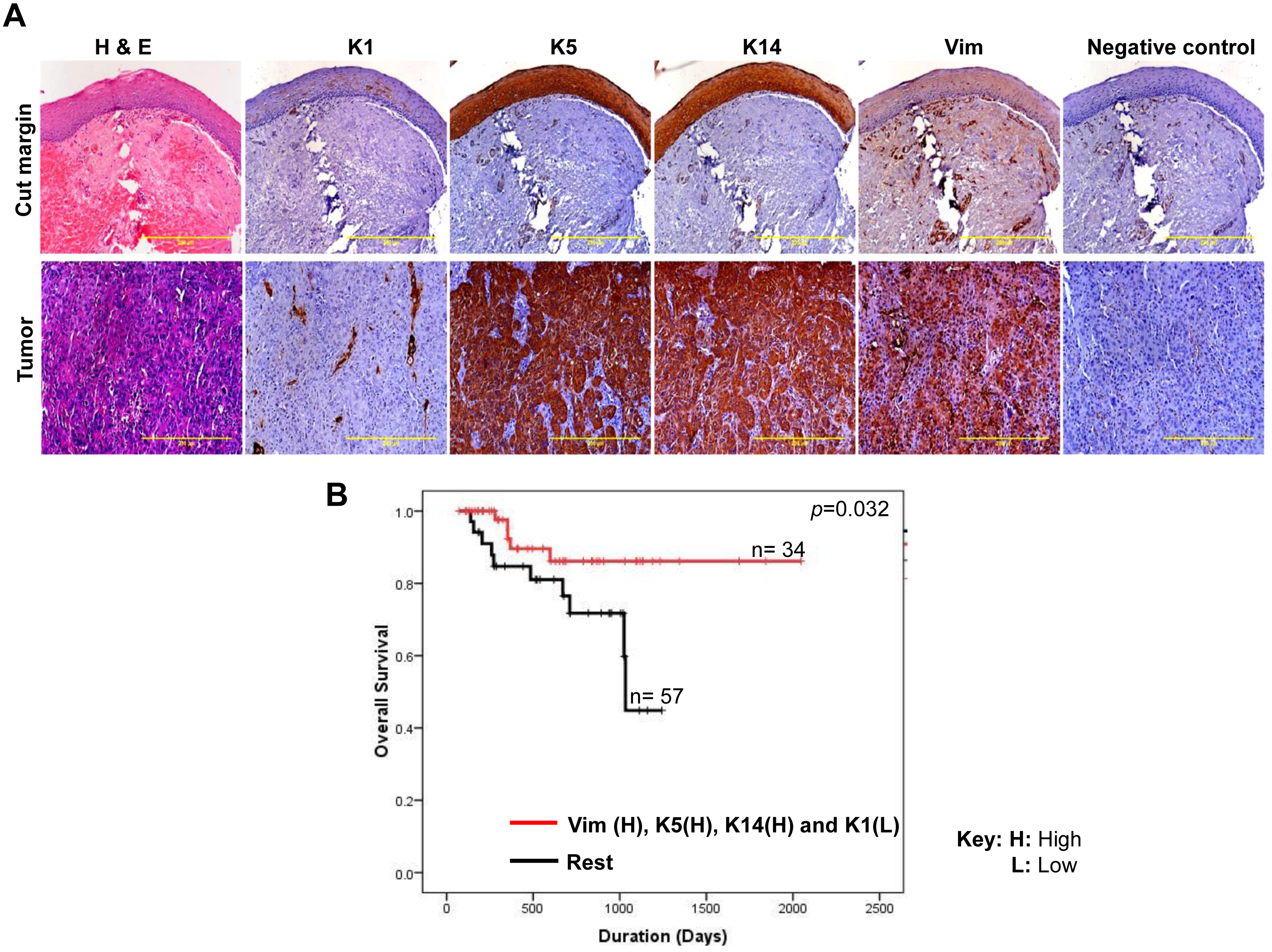

Supplement: S20 Fig — (A) The upper panel show images (Bar: 200μm) of IHC staining for K1, K5, K14 and vimentin expression in cut margin tissues while lower panel show images (Bar: 200μm) of IHC staining for K1, K5, K14 and vimentin expression in tumor tissues. The negative control images represent tissue sections incubated with serum from non-immunized mice in place of primary antibodies. (B) Kaplan-Meier survival analysis (n = 91) of high vimentin-K5-K14-low K1 expression vs. the other combinations of vimentin-K5-K14-K1 expression. (TIF) [file pone.0172559.s020.tif]

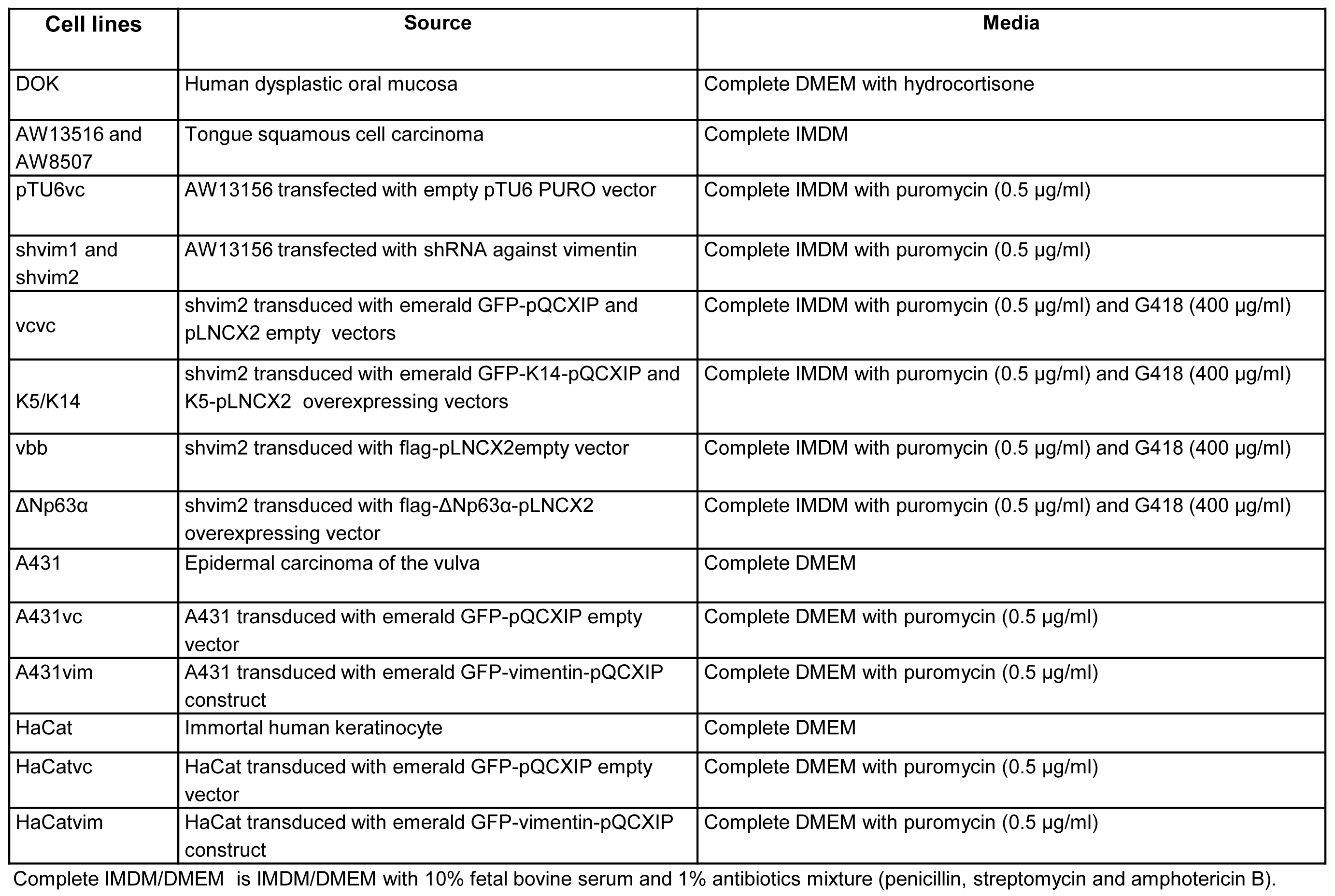

Supplement: S1 Table — The table shows a list of cell lines along with their particulars. (TIF) [file pone.0172559.s021.tif]

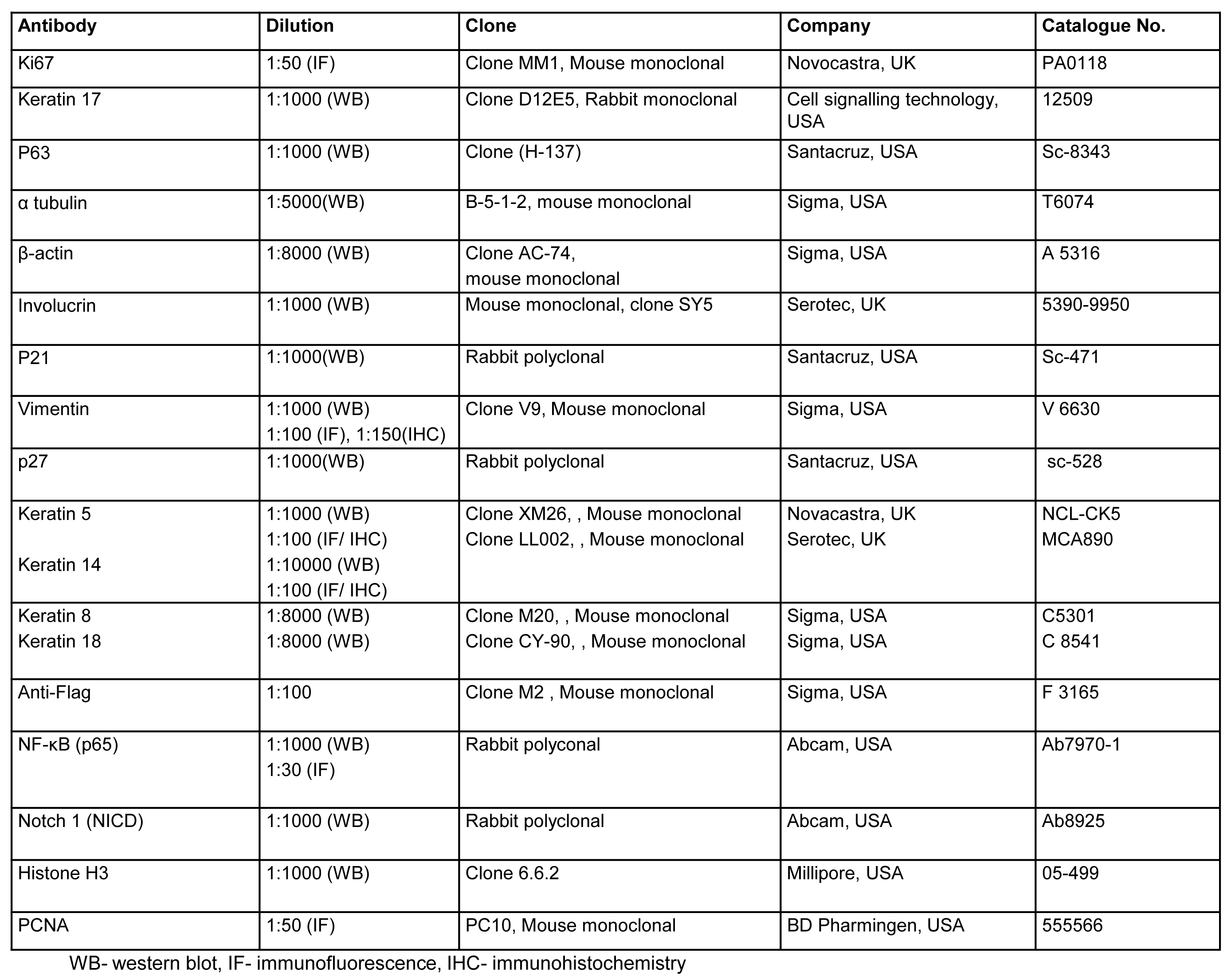

Supplement: S2 Table — The table shows a list of antibodies along with their particulars. (TIF) [file pone.0172559.s022.tif]

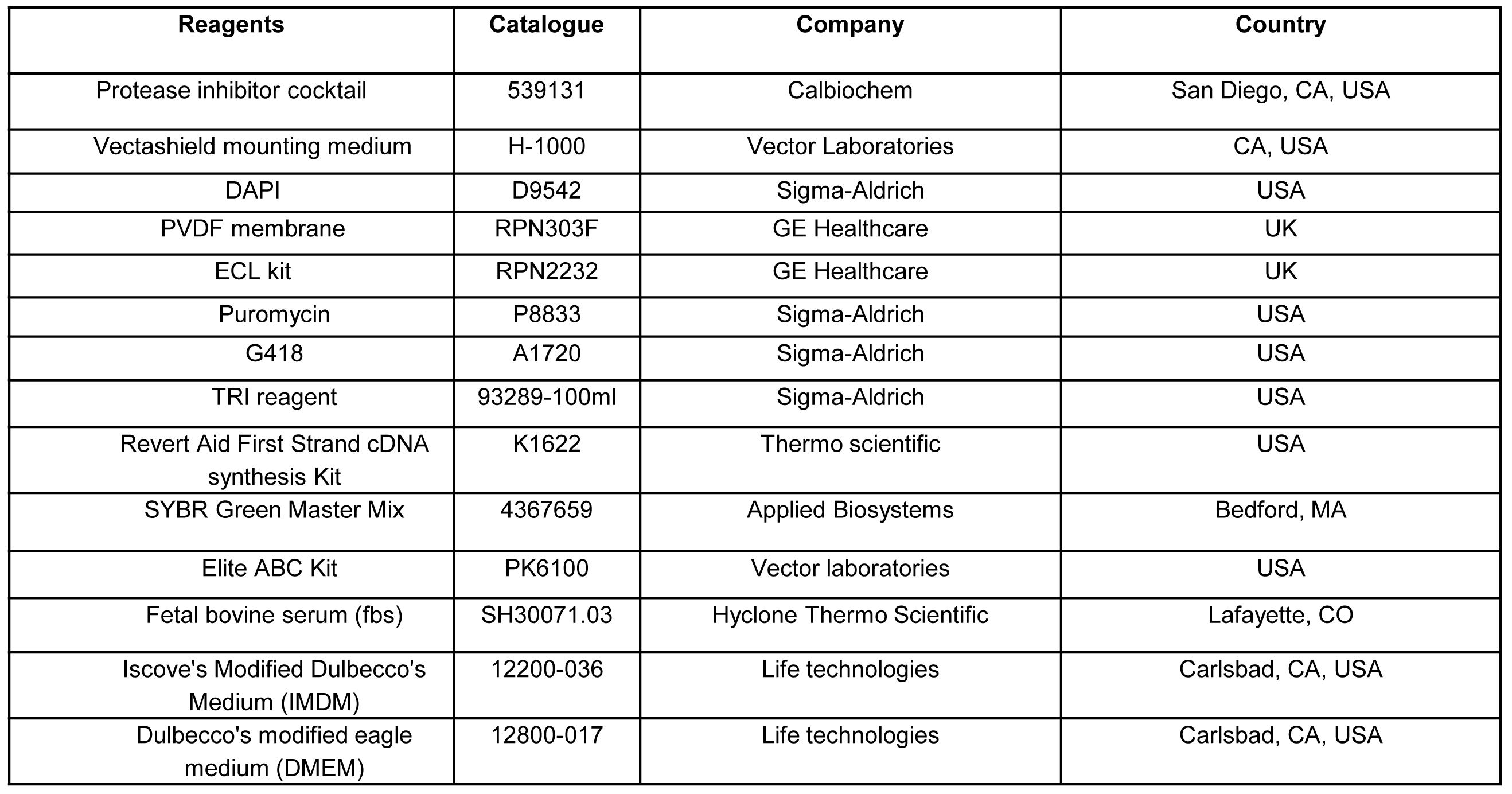

Supplement: S3 Table — The table shows a list of reagents along with their particulars. (TIF) [file pone.0172559.s023.tif]

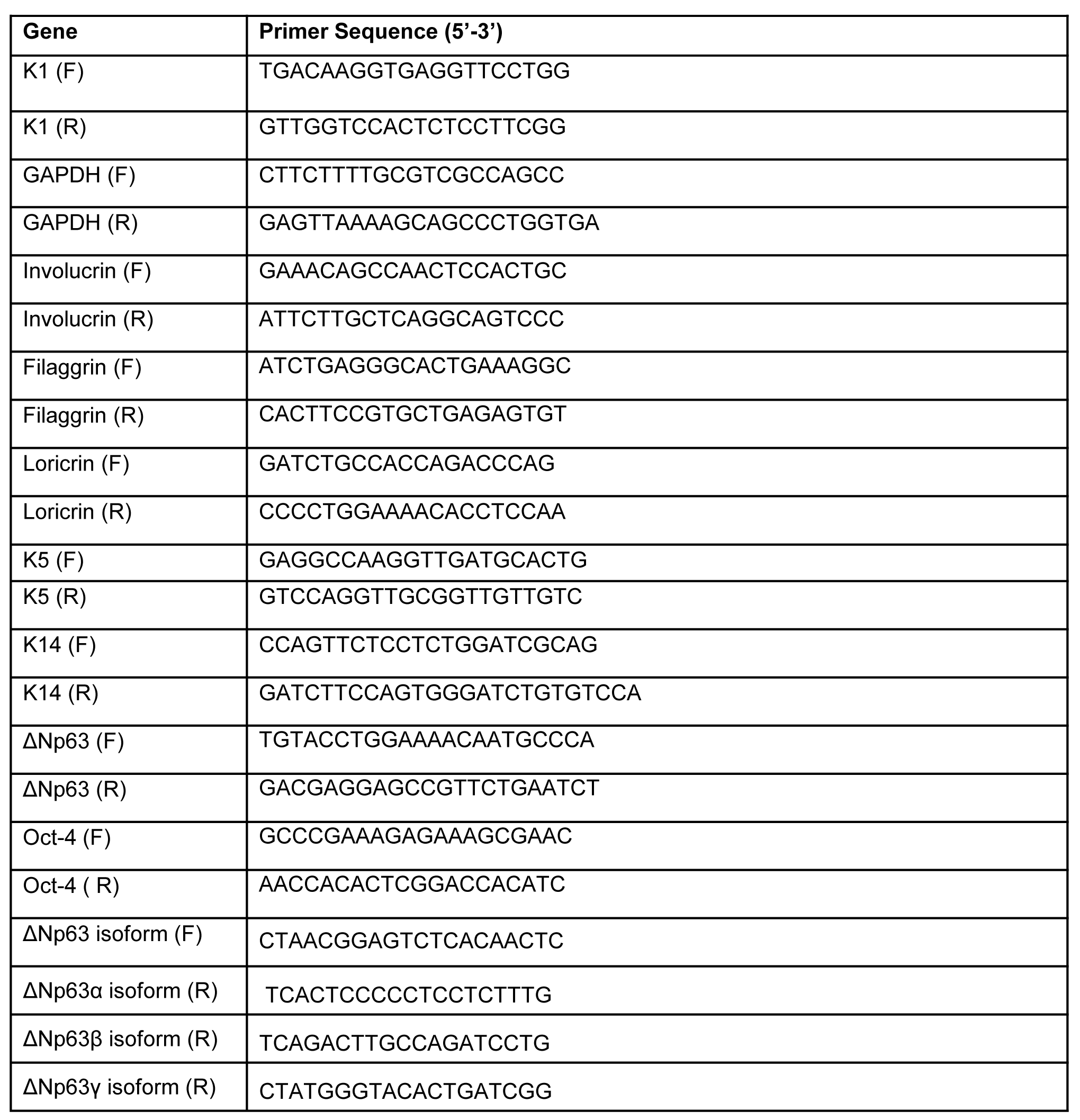

Supplement: S4 Table — The table shows a list of primer sequences used for RT-PCR and qRT-PCR analysis. (TIF) [file pone.0172559.s024.tif]

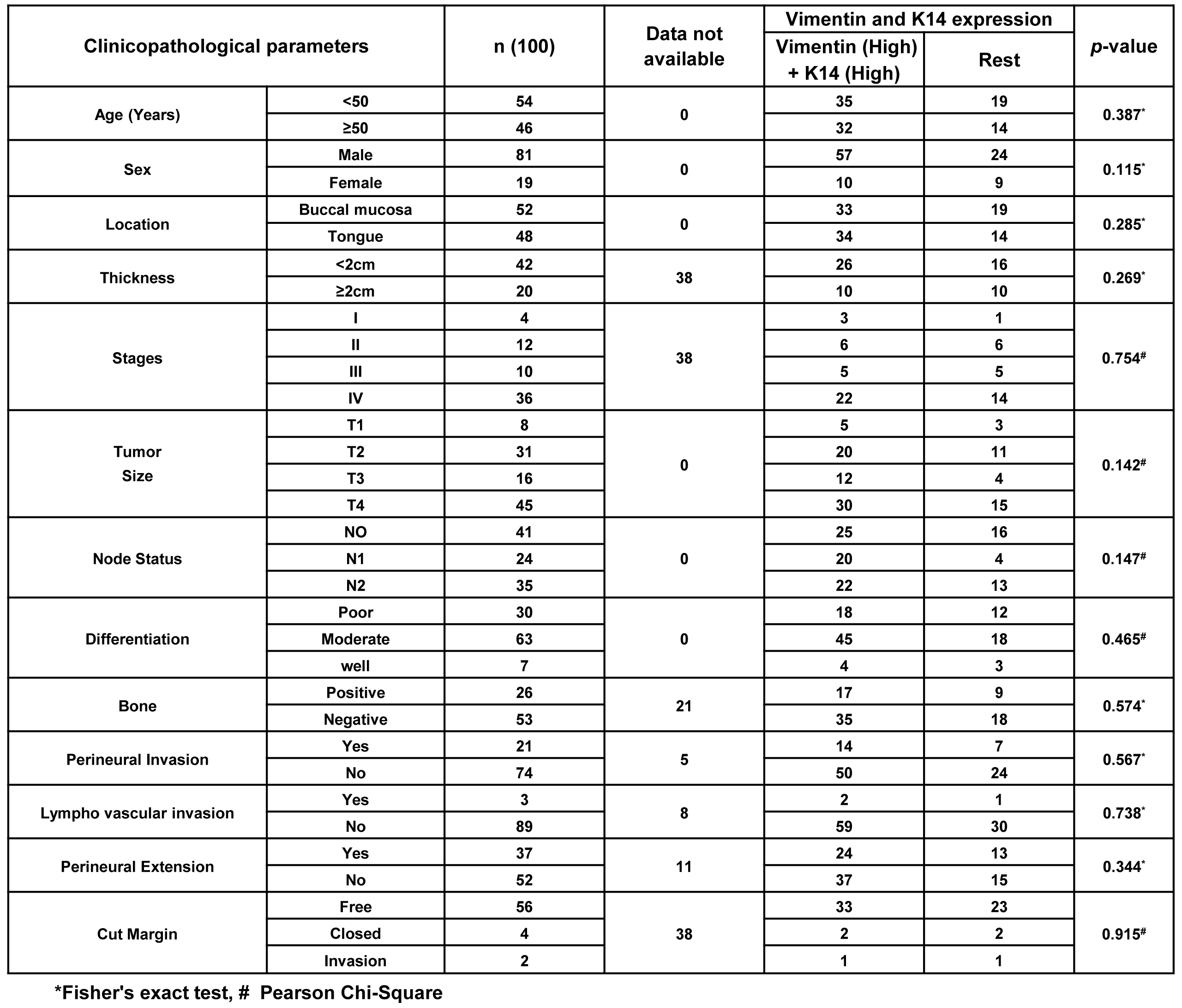

Supplement: S5 Table — (TIF) [file pone.0172559.s025.tif]

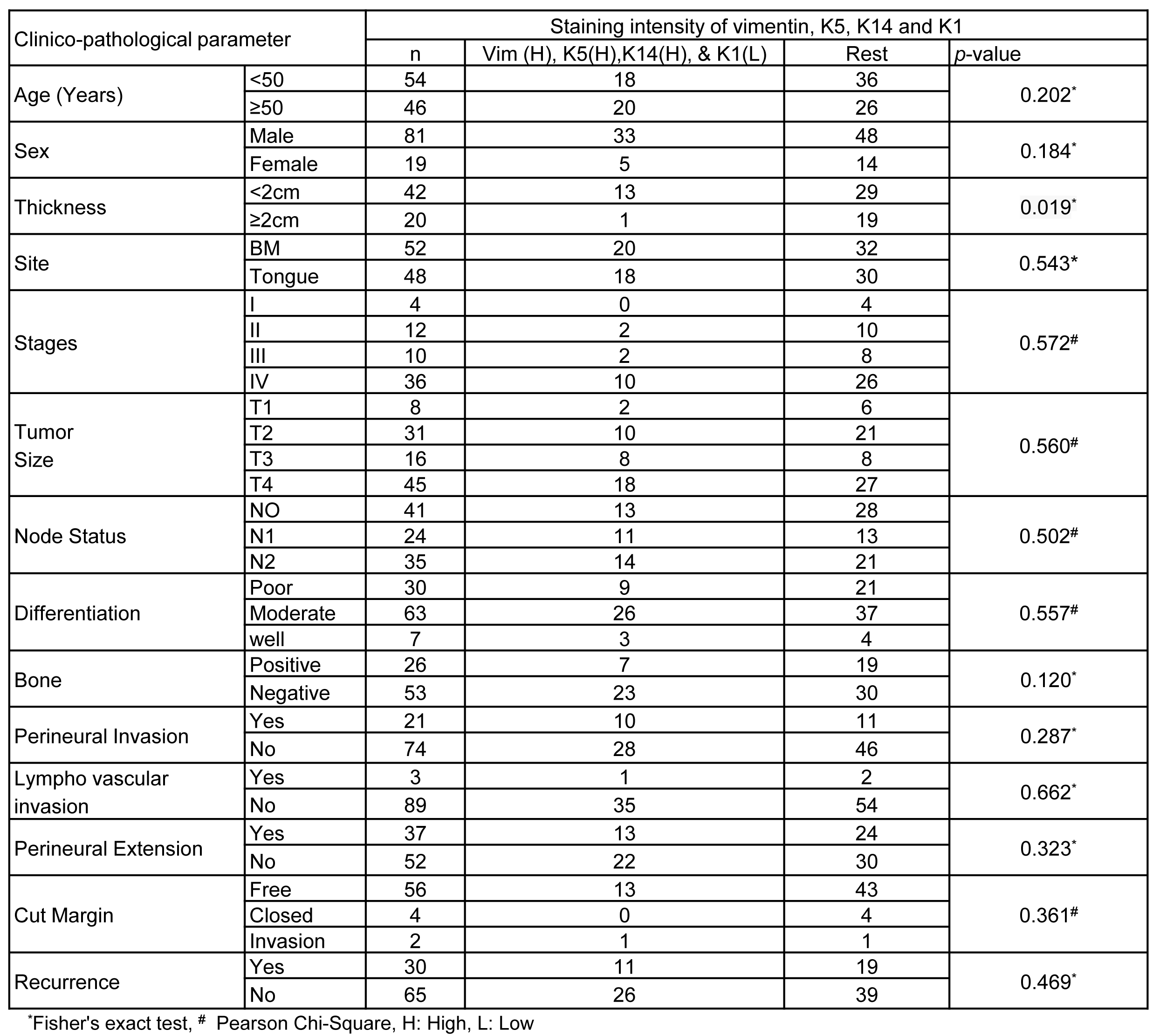

Supplement: S6 Table — (TIF) [file pone.0172559.s026.tif]
